# Structurally Diverse Sesquiterpenoids with Anti-neuroinflammatory Activity from the Endolichenic Fungus *Cryptomarasmius aucubae*

**DOI:** 10.1007/s13659-021-00299-9

**Published:** 2021-05-07

**Authors:** Yi-Jie Zhai, Jian-Nan Li, Yu-Qi Gao, Lin-Lin Gao, Da-Cheng Wang, Wen-Bo Han, Jin-Ming Gao

**Affiliations:** Shaanxi Key Laboratory of Natural Products and Chemical Biology, College of Chemistry and Pharmacy, Northwest A&F University, Yangling, Shaanxi 712100 People’s Republic of China

**Keywords:** Endolichenic fungus, *Cryptomarasmius aucubae*, Sesquiterpenes, Anti-neuroinflammatory activity

## Abstract

Two new sterpurane sesquiterpenoids named sterpurol D (**1**) and sterpurol E (**2**), and one skeletally new sesquiterpene, cryptomaraone (**3**), bearing a 5,6-fused bicyclic ring system, along with five known ones, sterpurol A (**4**), sterpurol B (**5**), paneolilludinic Acid (**6**), murolane-2*α*, 9*β*-diol-3-ene (**7**) and (–)-10,11-dihydroxyfarnesol (**8**) were isolated from an endolichenic fungus *Cryptomarasmius aucubae*. The structures of the new compounds were elucidated by analysis of NMR spectroscopic spectra and HRESIMS data. The absolute configurations of **1** and **2** were established by spectroscopic data analysis and comparison of specific optical rotation, as well as the biosynthetic consideration. Additionally, compounds **1**, **2**, **4**–**6**, and **8** showed significant nitric oxide (NO) production inhibition in Lipopolysaccharide (LPS)-induced BV-2 microglial cells with the IC_50_ values ranging from 9.06 to 14.81 *μ*M.

**Supplementary Information:**

The online version contains supplementary material available at 10.1007/s13659-021-00299-9.

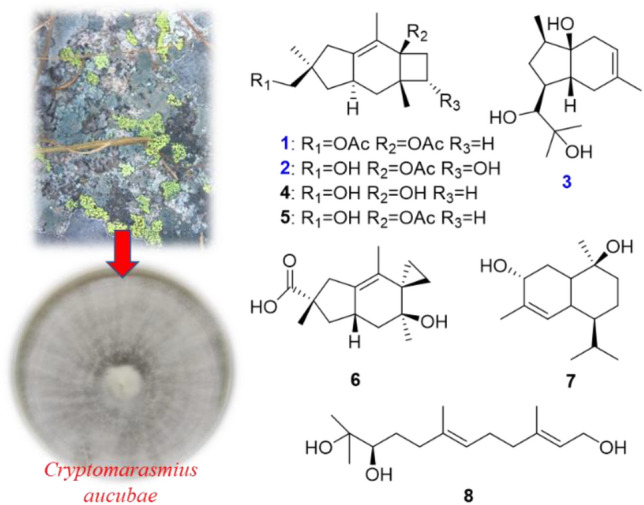

## Introduction

Endolichenic fungi parasitizing in the thalli of lichens bear resemblance to the endophytes residing in the tissues of higher plants [1–3]. In the past dozen years, endolichenic fungi have been considered as the promising bioresources owing to their ability to produce a variety of secondary metabolites, including alkaloids [4, 5], polyketides [6, 7], terpenoids [8, 9], xanthones [10], heptaketides [11], and cyclic peptides [12, 13], exhibiting a diverse array of biological activities, such as anticancer [11], antimicrobial [10], cytotoxic [14], antioxidant[15], anti-Alzheimer’s disease, and anti-inflammatory [16].

Thousands of sesquiterpenoids have been reported in the literature, however, the occurrence of sterpurane and illudane sesquiterpenoids are rare in nature [17]. Since the first discovery of sterpurane-type sesquiterpene, sterpuric acid, from *Stereum purpureum* in 1981 [18], many kinds of these compounds have been characterized in succession from the basidiomycetes *Merulius tremellosus*, *Phlebia tremellosa* o*r Phlebia uda* [19–21], *Clavicorona pyxidata* [22], *Flammulina velutipesin* [23], and *Gloeophyllum* sp. [24], as well as from *Phlebia* spp. and the soft coral *Alcyonium acaule* [25].

**Fig. 1:**
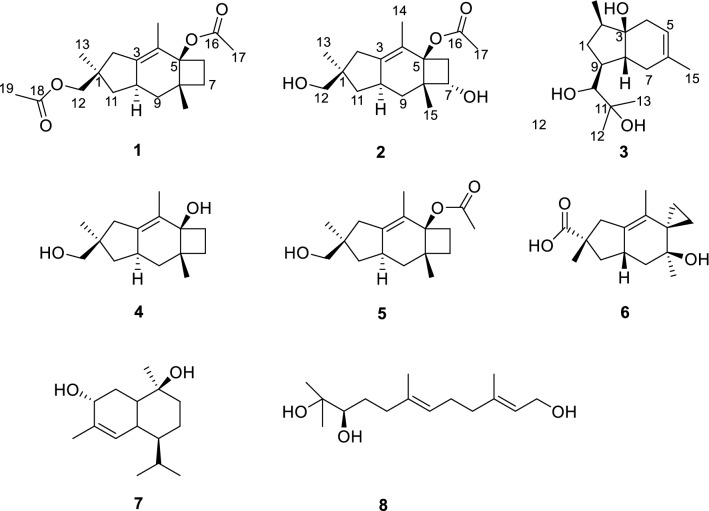
Structures of compounds **1**–**8**

In continuation of our research on new and/or bioactive secondary metabolites from the endophytic fungi [26–28], a lichen-forming fungus *Cryptomarasmius aucubae* was isolated from the lichen collected from Hua Mountain in Shaanxi Province. After the cultivation of this fungus in cooked rice medium, eight sesquiterpenes (**1**–**8**) (Fig. 1), including three unreported and five known compounds, were obtained. Among them, compounds **2** and **5** were demonstrated to be potent anti-neuroinflammatory agents in lipopolysaccharide (LPS)-induced BV-2 microglial cells with the IC_50_ values of 9.93 and 9.06 *μ*M, respectively, which were comparable to that of quercetin (IC_50_ = 9.75 *μ*M**)** used as a positive control**.** Herein, the details of isolation, structure elucidation, and anti-neuroinflammatory activities of these compounds are presented.

## Results, Discussion and Conclusion

**Fig. 2:**
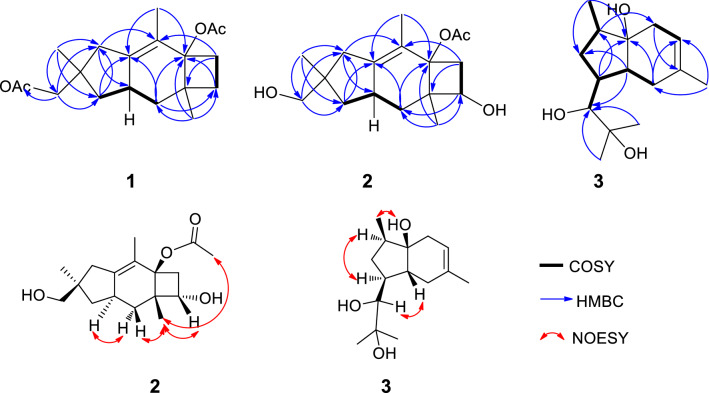
Key COSY, HMBC and NOESY correlations of **1**–**3**

The molecular formula of **1** was established to be C_19_H_28_O_4_, six degrees of unsaturation, on the basis of the HRESIMS at *m/z* 343.1876 [M + Na]^+^ (calcd for C_19_H_28_O_4_Na, 343.1880). The ^1^H spectrum of **1** was very similar to that of the coexisting known sterpurol B (**5**), the only difference between them was that the hydrogen atom on the 12-OH in **5** was replaced by an acetyl group in **1** at *δ*_H_ 1.95 and *δ*_C_ 20.8/171.2 (Tables 1, 2). This indicated that **1** was an acetylated derivative of **5**. In the HMBC spectrum of **1** (Fig. 2), the correlations from H-7 to C-5, C-6, C-8 and C-15, from H-9 to C-3, C-5, C-7, C-8 and C-10, from H-12 to C-1, C-2, C-11, C-13 and C-18, from CH_3_-13 to C-1, C-2, C-11 and C-12, from CH_3_-14 to C-3, C-4, C-5, C-8 and C-10, and from CH_3_-15 to C-5, C-7, C-8 and C-9, established the planar structure of **1** as a sterpurane-type sesquiterpene with two acetyl groups attached at C-5 and C-12. The absolute configuration of **1** (1*R*, 5*R*, 8*S*, 10*R*) was evidenced to be identical with that of **5**, due to the same optical rotation for **1** ([*α*] ^25^D + 26.4 (*c* 0.05, MeOH)) as that for **5** ([*α*] ^25^D + 22.3 (*c* 0.05, MeOH)). Thus, the structure of **1** was determined as shown in Fig. 1.

**Table 1:** ^1^H NMR data (*δ* in ppm, *J* in Hz) of compounds **1**–**3**

No.	**1**^a^ *δ*_H_, mult (*J*)	**2**^b^ *δ*_H_, mult (*J*)	**3**^b^ *δ*_H_, mult (*J*)
1			1.96 (m)
			1.05 (m)
2	2.28 (d, 17.0)	2.19 (d, 17.0)	1.52 (ddd, 9.7, 6.7, 2.8)
	2.10 (d, 17.3)	2.08 (d, 17.0)	
3			
4			2.24 (d, 17.5)
			1.91 (m)
5			5.30 (s)
6	1.90 (ddd, 11.3, 9.0, 2.3)	2.46 (dd, 11.5, 7.5)	
	2.36 (q, 10.5)	2.26 (dd, 11.5, 8.3)	
7	1.68 (q,10.1)	3.91 (t, 7.9)	2.15 (dd, 16.5, 6.6)
	1.29 (td, 10.8, 2.3)		1.95 (d, 16.7)
8			1.18 (m)
9	1.36 (t, 11.9)	1.40 (dd, 13.0,11.3)	1.65 (m)
	1.47 (t, 11.9)	1.71 (dd,13.0, 6.4)	
10	2.65 (m)	2.53 (m)	3.34 (d, 10.2)
11	1.98 (m)	1.96 (dd, 12.4, 7.5)	
	1.12 (m)	1.15 (d, 11.9)	
12	3.98 (d, 10.8)	3.48 (d, 10.8)	1.16 (s)
	3.86 (d, 10.8)	3.40 (d, 10.6)	
13	1.08 (s)	1.08 (s)	1.22 (s)
14	1.48 (s)	1.48 (s)	0.96 (s)
15	1.17 (s)	1.12 (s)	1.69 (s)
16			
17	2.04 (s)	2.01 (s)	
18			
19	1.95 (s)		

^a^Recorded at 500 MHz, recorded in acetone-*d*_6_

^b^Recorded at 500 MHz, recorded in CDCl_3_

**Table 2:** ^13^C NMR data (*δ* in ppm) of compounds **1**–**3**

No.	**1**^a^ *δ*_C_, type	**2**^b^ *δ*_C_, type	**3**^b^ *δ*_C_, type
1	41.3, C	42.2, C	28.3, CH_2_
2	40.8, CH_2_	39.7, CH_2_	42.6, CH
3	139.7, C	139.7, C	72.4, C
4	125.3, C	124.2, C	35.4, CH_2_
5	81.1, C	76.2, C	118.5, CH
6	32.4, CH_2_	43.1, CH_2_	134.0, C
7	22.9, CH_2_	63.1, CH	27.0, CH2
8	44.9, C	50.4, C	30.7, CH
9	35.7, CH_2_	33.5, CH_2_	30.3, CH
10	37.4, CH	36.7, CH	79.6, CH
11	43.7, CH_2_	42.6, CH_2_	73.1, C
12	71.4, CH_2_	70.5, CH_2_	23.3, CH_3_
13	25.9, CH_3_	25.1, CH_3_	26.5, CH_3_
14	13.0, CH_3_	12.9, CH_3_	14.2, CH_3_
15	23.7, CH_3_	16.0, CH_3_	23.2, CH_3_
16	171.2, C	169.9, C	
17	20.9, CH_3_	21.0, CH_3_	
18	169.5, C		
19	20.8, CH_3_		

^a^Recorded at 125 MHz, recorded in acetone-*d*_6_

^b^Recorded at 125 MHz, recorded in CDCl_3_

Compound **2** have the molecular formula of C_17_H_26_O_4_ (five degrees of unsaturation) on the basis of HR-ESI-MS at *m/z* 317.1724 [M + Na]^+^ (calcd for C_17_H_26_O_4_Na, 317.1723). Its ^1^H and ^13^C NMR spectra (Tables 1 and 2) were similar to those of** 5** except for a hydroxyl group (*δ*_H_ 3.91; *δ*_C_ 63.1). The location of the hydroxyl group at C-7 in **2** was determined by the observation of HMBC correlations from H-7 to C-6, C-8, C-9 and C-15. Detailed analysis of HSQC and HMBC spectra confirmed the structure of **2** as shown in Fig. 1. Furthermore, the relative configuration of **2** was determined by analysis of NOESY data. The obvious NOESY correlations (Fig. 2) of H-7 with H-15, H-15 with H_a_-9 (*δ*_H_ 1.40) and H-17, indicated that they were all positioned on the same face of the tricyclic structure. In addition, the correlations of H-10 with H_b_-9 (*δ*_H_ 1.71) indicated they were opposite orientation. The absolute configuration of **2** was determined using the modified Mosher's method [29] but failed, due to the instability of the sample. Nevertheless, based on the consideration of the biogenesis, the absolute configuration of **2** was deduced to be identical to that of **5**, and was thus determined as 1*R*, 5*R*, 7*S*, 8*S*, 10*R*.

Compound **3** has a molecular formula of C_15_H_26_O_3_ as determined by the HR-ESI-MS at *m/z* 277.1775 [M + Na]^+^ (Calcd for C_15_H_26_O_3_Na 277.1774), three degrees of unsaturation. The ^1^H-NMR spectrum of **3** displayed resonances for one doublet and three singlet methyls at *δ*_H_ 0.96 (3H, d, *J* = 6.9 Hz), 1.16 (3H, s), 1.22 (3H, s), 1.69 (3H, s), an olefnic proton at *δ*_H_ 5.30 (1H, s), an oxymethine at *δ*_H_ 3.34 (1H, d, *J* = 10.2 Hz) and other signals for aliphatic protons (Tables 1 and 2). The ^13^C-NMR and HSQC spectrum displayed 15 resonances including two olefinic carbons at *δ*_C_ 118.5 and 134.0, one oxymethine signal at *δ*_C_ 79.6, two quaternary carbons bearing hydroxyl groups at *δ*_C_ 72.4 and 73.1, four methyls at *δ*_C_ 14.2, 23.2, 23.3, 26.5, three methylenes at *δ*_C_ 27.0, 28.3, 35.4, three methines at *δ*_C_ 30.3, 30.7, 42.6. Analysis of the HMBC spectrum of **3**, the correlations from H-12 to C-10, C-11 and C-13, from H-13 to C-10, C-11 and C-12, from CH_3_-14 to C-1, C-8 and C-9, and from CH_3_-15 to C-3, C-4 and C-5, demonstrated the presence of the chain (2-methylpropane-1,2-diol) located at C-9 of the bicarbocyclic ring moiety. The NOESY spectrum was measured in MicroCryoProbe (DMSO-*d*_6_, Fig. S19 in the Supporting Information). NOEs of 3-OH with H_a_-1 and H-14, H-2 with H_b_-1 and H-9, H-8 with H-10 assigned its relative configuration as shown (Fig. 2), however, the absolute configuration of **3** was not assigned due to the paucity of the sample. To the best of our knowledge, this is the first report of 5,6-fused bicyclic natural sesquiterpene with the 2-methylpropane-1,2-diol moiety anchored to the cyclopentane ring.

The structures of the remaining known compounds were identified as sterpurol A (**4**), sterpurol B (**5)** [30], an illudane sesquiterpene paneolilludinic acid (**6**) [31], the plant cadinane sesquiterpenoid murolane-2*α*, 9*β*-diol-3-ene (**7**) [32], and (–)-10,11-dihydroxyfarnesol (**8**) [33] by comparison of their NMR data with those in the literature.

All of the compounds were tested for their anti-inflammatory activities by restraining the production of NO in lipopolysaccharide (LPS)-induced BV-2 microglial cells (Table 3). As a result, compounds **1**, **2**, **4**, **5**, **6**, **7** and** 8** exhibited 75.9, 85.4, 73.1, 99.3, 79.1, 51.7 and 76.8% inhibition at 20 *μ*M, respectively, whereas the positive control quercetin showed 95.6% inhibition at 20 *μ*M. As shown in Table 3, the isolated compounds (except for **7**) exhibited inhibitory effect with IC_50_ values ranging from 9.06 to 14.81 *μ*M, of which **5** was the most active compound with the IC_50_ value of 9.06 *μ*M. In addition, in vitro these sesquiterpenes were also assayed for other bioactivities, such as *α*-glucosidase inhibition, and antibacterial, however, they were inactive.

**Table 3:** Inhibitory effects of compounds **1**–**8** on NO production induced by LPS in BV-2 microglial cells

Compound	IC_50_ (*μ*M)	Cell viability^a^ (%)
**1**	14.81 ± 2.23	103.46 ± 4.73
**2**	9.93 ± 0.99	97.07 ± 4.0
**3**	NT	NT
**4**	15.32 ± 1.43	98.72 ± 1.18
**5**	9.06 ± 1.13	106.83 ± 2.73
**6**	11.49 ± 0.58	104.05 ± 2.67
**7**	> 20	NT
**8**	12.17 ± 0.40	99.12 ± 0.18
Quercetin^b^	9.75 ± 0.79	101.54 ± 0.83

^a^Cell viability was expressed as a percentage (%) of that the LPS-only treatment group

^b^Positive control. NT was not texted

In summary, eight secondary metabolites, including three new sesquiterpenoids, sterpurols D (**1**) and E (**2**), and cryptomaraone (**3**), and five known sesquiterpenes (**4–8**) were isolated and identified from the endolichenic fungus *C. aucubae* in rice solid-substrate fermentation. Compound **5** showed significant anti-inflammatory activity by reducing the release of NO in LPS-induced BV-2 Microglial cells without cytotoxicity at 50 *μ*M. Besides, compounds **1, 2, 4, 6** and** 8** displayed moderate anti-inflammatory activity. These findings are of value in searching for new anti-neuroinflammatory agents.

## Experimental Section

### General Method

Infrared (IR) spectra were recorded on a Bruker Tensor 27 spectrophotometer (Bruker Optics, Rheinstetten, Germany) with KBr pellets. Ultraviolet (UV) measurements were obtained using an ultraviolet–visible (UV–vis) Evolution 300 spectrometer (Thermo Fisher Scientific, Inc., Waltham, MA, USA). High-resolution electrospray ionization mass spectrometry (HRESIMS) spectra were performed on an Agilent 6210 TOF LC-MS instrument equipped with an electrospray ionization (ESI) probe operating in positive-ion mode with direct infusion. Optical rotations were measured on an Autopol III automatic polarimeter (Rudolph Research Analytical, NJ, USA). Nuclear magnetic resonance (NMR) spectra were acquired on a Bruker Avance III 500 spectrometer (Bruker BioSpin, Rheinstetten, Germany), with tetramethylsilane (TMS) as an internal standard at room temperature. Silica gel (300–400 mesh, Qingdao Marine Chemical, Ltd., China), RP-18 gel (ODS-AQ-HG GEL, AQG12S50, YMC, Co., Ltd., Japan), and Sephadex LH-20 (GE Healthcare, Inc., Uppsala, Sweden) were used for column chromatography (CC). Fractions were monitored by thin-layer chromatography (TLC) (Huanghai Marine Chemical, Ltd., China). Semi-preparative reversed-phase high-performance liquid chromatography (RP-HPLC) were analyzed by an Aligent 1100 (Agilent Technologies, Inc., California, USA) liquid chromatography system equipped with a Aligent C18 column (EclipseXDB-C18, 5 µm, 9.4 × 250 mm). The *α*-glucosidase inhibitory assay was measured by a microplate reader (Synergy HTX, BioTek Instruments Inc., Winooski, VT, USA). All other chemicals used in this study were of analytical grade.

### Fungal Material

The fungus, isolated from the crustose lichen collected in Hua Mountain, Huayin county, Shaanxi Province, China, in May 2017, was identified as *Cryptomarasmius aucubae* based on the DNA sequencing of the ITS of rDNA (GenBank: NO. MW174800). The strain was assigned the accession No. SF69 and deposited in the Shaanxi Key Laboratory of Natural Products and Chemical Biology, Northwest A&F University, Yangling, China.

### Fermentation and Extraction

The strain was activated by potato dextrose agar (PDA) medium in plates at 28 °C for 5 days. Then, the well-grown plate of the strain was cut into small pieces with a size of about 5 mm^2^, and the small pieces were inserted into 1000 mL Erlenmeyer flasks each containing 400 mL of potato dextrose (PD) liquid medium for culturing. The seed liquids were cultivated at 28 °C for 3 days on a shaking table at 120 rpm. Next, 20 mL seed liquid was poured into a rice medium (40 g rice, 60 mL distilled water) in 150 Erlenmeyer flasks (500 mL). After the fungi were fermented at 28 °C for 42 days, cultures were extracted two times with methanol. The methanol extract was vacuum filtered and dried under reduced pressure to yield a crude extract. The extract was dissolved and extracted with ethyl acetate and water in the volume ratio of 1:1 (4 L) for three times, and combined the organic layer, then it was concentrated under reduced pressure to give a crude extract (25.7 g).

### Isolation of Metabolites **1**–**8**

Total sample was separated over a silial gel column to yield seven fractions with CHCl_3_-MeOH (v/v, 100:0 → 0:100, 3 L each). Fraction A was separated on Sephadex LH-20 eluted with MeOH and further purified by a RP-18 column eluted with a gradient of MeOH-H_2_O (v/v, 30 → 100%) to obtain one fraction A-1. Fraction A-1 was next purified by RP-HPLC with MeCN-H_2_O (72:28) to afford compound **1** (*t*_R_ = 28 min, 10.2 mg). Fraction C was separated by Sephadex LH-20 with MeOH to obtain Fraction C-1, and further purified by RP-HPLC with MeCN-H_2_O (55:45) to give compound **5** (*t*_R_ = 26 min, 8.5 mg). Fraction D was applied to a reversed phase C-18 column using MeOH-H_2_O (v/v, 30 → 100%) as solvent system and next separated by Sephadex LH-20 with MeOH to give Fraction D-1, and further purified by RP-HPLC with MeCN-H_2_O (42:58) to give compound **6** (*t*_R_ = 13 min, 4.3 mg) and compound **4** (*t*_R_ = 15 min, 15.4 mg). Fraction E was separated by a RP-18 column eluted with MeOH-H_2_O (v/v, 30 → 100%), followed by Sephadex LH-20 using MeOH and then purified by a RP-18 column eluted with MeOH-H_2_O (v/v, 50 → 100%) to gain Fraction E-1 and E-2. Fraction E-1 was further purified by RP-HPLC with MeCN-H_2_O (28:72) to afford compound **2** (*t*_R_ = 30 min, 10.7 mg). Fraction E-2 was subjected to column chromatography over reversed-phase silica gel eluted with MeOH-H_2_O (v/v, 50 → 100%) to obtain Fraction E-2-1, and further purified by RP-HPLC with MeCN-H_2_O (45:55) to yield compound **7** (*t*_R_ = 24 min, 13.5 mg). Fraction F was subjected to Sephadex LH-20 eluted with MeOH, then separated by a RP-18 column eluted with MeOH-H_2_O (v/v, 30 → 100%) and further purified by RP-HPLC with MeCN-H_2_O (25:75) to afford compound **8** (*t*_R_ = 47 min, 6.3 mg). Fraction G was separated by a RP-18 column eluted with MeOH-H_2_O (v/v, 30 → 100%), purified by Sephadex LH-20 using MeOH, and further separated by RP-HPLC with MeCN-H_2_O (30:70) to yield compound **3** (*t*_R_ = 20 min, 2.1 mg).

### Spectroscopy Data of Compounds

Sterpurol D (**1**): Colorless solid; [*α*]_D_^25^ + 26.4 (*c* 0.05, MeOH); UV (MeOH) *λ*_max_ (log *ε*) 230 (3.63); IR (KBr) *ν*_max_ 3470, 2950, 2313, 1738, 1454, 1375, 1241, 1150, 1033, 647, 605 cm^−1^; ^1^H and ^13^C NMR data, see Table 1; HR-ESI-MS *m/z* 343.1876 [M + Na]^+^ (calcd. for C_19_H_28_O_4_Na, 343.1880).

Sterpurol E (**2**): Colorless solid; [*α*]_D_^25^ + 758.8 (*c* 0.05, MeOH); UV (MeOH) *λ*_max_ (log *ε*) 234 (3.34); IR (KBr) *ν*_max_ 3388, 2933, 2871, 2316, 1726, 1451, 1373, 1246, 1121, 1026, 916, 792, 606 cm^−1^; ^1^H and ^13^C NMR data, see Table 1; HR-ESI-MS *m/z* 317.1704 [M + Na]^+^ (calcd. for C_17_H_26_O_4_Na, 317.1723).

Cryptomaraone (**3**): Colorless solid; [*α*]_D_^25^ – 47.8 (*c* 0.05, MeOH); ^1^H and ^13^C NMR data, see Table 1; HR-ESI-MS *m/z* 277.1775 [M + Na]^+^ (calcd. for C_15_H_26_O_3_Na, 277.1774).

Sterpurol B (**5**): Colorless solid; [*α*]_D_^25^ + 22.3 (*c* 0.05, MeOH); ^1^H and ^13^C NMR data, see Figs. S22 and S23; HR-ESI-MS *m/z* 301.1775 [M + Na]^+^ (calcd. for C_17_H_26_O_3_Na, 301.1774).

### Cell Viability Was Evaluated By MTT Assay

BV-2 murine microglial cells, acquired from Peking Union Medical College Cell Bank, were cultured in Dulbecco’s modified Eagle’s medium supplemented with 10% (v/v) heat-inactivated fetal bovine serum, penicillin (100 U/mL), and streptomycin (100 U/mL) in carbon dioxide cell incubator. When cell growth density outnumbered 90%, BV-2 cells were seeded in 96-well plates at a density of 2 × 10^4^/well, 100 *μ*L) and incubated for 24 h. Next, the cells were treated with the compounds (DMSO as solvent) at 20 *μ*M for 24 h in DMEM with 1 *μ*g/mL LPS. Cells treated with DMSO alone were used as the negative control. After adding 20 *μ*L of 10 mg/mL MTT reagent to each well, the samples were shaken lightly and incubated at 37 °C for 4 h. The supernatant was removed, the blue-purple crystals were fully dissolved in DMSO (200 *μ*L), and the absorbance of each well was read at 570 nm (Tecan Sunrise, Switzerland) [26, 34]. Percentage of cell viability is calculated as: (absorbance of treated well/absorbance of control well) × 100%.

### Nitric Oxide (NO) Production Inhibitory Assay

BV-2 cells were seeded into 96-well plates at 2 × 10^4^ cells/100 *μ*L of medium and incubated for 24 h. Then, cells were treated with 1 *μ*g/mL of lipopolysaccharide (LPS) and various concentrations (0.1–20.0 *μ*M) of test compounds (DMSO as solvent) for 24 h. An equal amount of DMSO and LPS were served as the controls; quercetin (J&K Scientific, Beijing, China) was taken as the positive control). The NO concentration in the medium was measured by using a Nitric Oxide Assay Kit, according to the accumulated levels of nitrite in the supernatants by a standard Griess reaction [26, 34]. As follows, 50 *μ*L of the culture supernatant of BV-2 cells was reacted with 50 *μ*L of Griess reagent I and Griess regent II successively in a 96-well plate. The absorbance at 570 nm of the mixture was measured using a microplate reader. IC_50_ values were calculated as the concentrations that reduced NO production by 50%. Quercetin was taken as the positive control.

### *α*-Glucosidase Inhibitory Assay

*α*-Glucosidase Inhibitory assay was tested following the methods reported previously [35, 36] with slight modification. The assay mixture (720 *μ*L) contained 572.4 *μ*L of 0.05 M phosphate buffer (pH 6.8), 3.6 *μ*L of enzyme solution (10 U/mL), and 36 *μ*L of 0.4 mM inhibitors (the tested compounds, genistein as positive control) were incubated at 37 °C for 10 min. Subsequently, 108 *μ*L of 6 mM *p*NPG (4-nitrophenyl *α*-d-glucopyranoside) was added to the preincubated solutions, and the mixtures were incubated at 37 °C for 40 min. Then absorbance of the mixture at 405 nm was recorded. The negative control was prepared by adding PBS instead of *α*-glucosidase, the blank was prepared by adding solvent instead of tested compounds, and the inhibition rate was calculated as the following equation:

$$\frac{{\left( {{\text{OD}}_{{{\text{control}}}} - {\text{OD}}_{{\text{control blank}}} } \right) - \left( {{\text{OD}}_{{{\text{test}}}} - {\text{OD}}_{{\text{test blank}}} } \right)}}{{{\text{OD}}_{{{\text{control}}}} - {\text{OD}}_{{\text{control blank}}} )}} \times 100\% .$$

### Antibacterial Assay

Antibacterial activities were evaluated according to the previously published report [37] with slight modification. Compounds **1** − **8** were tested in vitro for antibacterial activity against nine bacteria (*Escherichia coli*, *Bacillus subtilis*, *Staphylococcus aureus*, *Bacillus cereus*, *Erwinia carotovora* pv.*caratovora*, *Pseudomonas syringae*, *Erwinia carotovora subsp. Carotovora* and *Ralstonia solanacearum*). The tested bacteria were incubated in the beef extract-peptone medium (BPA) at 30 °C at 120 rpm for 12 h and the spore concentration was diluted to approximately 2 × 10^6^ CFU/mL with BPA medium. 50 *µ*L of suspension was added to 96-well microplates, then 50* µ*L of compounds (Ampicillin and streptomycin as positive control) dissolved in DMSO-BPA medium was added to give a final concentration of 100* µ*M. After incubation at 30 °C for 24 h, the absorbance of the mixture at 600 nm was recorded.

## Supplementary Information

Below is the link to the electronic supplementary material.

Supplementary file 1 (DOCX 4069 KB)
