# Supplementary material for: Structurally Diverse Sesquiterpenoids with Anti-neuroinflammatory Activity from the Endolichenic Fungus Cryptomarasmius aucubae
Source: Nat Prod Bioprospect. 2021 May 7;11(3):325–32. doi: 10.1007/s13659-021-00299-9 (PMC8141073; doi:10.1007/s13659-021-00299-9)
Supplement: Supplementary file 1 — (DOCX 4069 KB) [file 13659_2021_299_MOESM1_ESM.docx]

**Supporting Information**

Diverse sesquiterpenes with anti-neuroinflammatory activity from the Endolichenic Fungus *Cryptomarasmius aucubae*

Yi-Jie Zhai, Jian-Nan Li, Lin-Lin Gao, Da-Cheng Wang, Wen-Bo Han* and Jin-Ming Gao*

Shaanxi Key Laboratory of Natural Products & Chemical Biology, College of Chemistry & Pharmacy, Northwest A&F University, Yangling, Shaanxi 712100, People’s Republic of China, e-mail: jinminggao@nwsuaf.edu.cn

**Table of contents**

Figure S1. The ^1^H NMR spectrum of **1** in acetone-*d*_6_ (500 MHz).

Figure S2. The ^13^C NMR spectrum of **1** in acetone-*d*_6_ (125 MHz).

Figure S3. The HSQC spectrum of **1** in acetone-*d*_6._

Figure S4. The ^1^H−^1^H COSY spectrum of **1** in acetone-*d*_6._

Figure S5. The HMBC spectrum of **1** in acetone-*d*_6._

Figure S6. The ^1^H NMR spectrum of **2** in CDCl_3_ (500 MHz).

Figure S7. The ^13^C NMR spectrum of **2** in CDCl_3_ (125 MHz).

Figure S8. The HSQC spectrum of **2** in a CDCl_3._

Figure S9. The ^1^H−^1^H COSY spectrum of **2** in CDCl_3._

Figure S10.The HMBC spectrum of **2** in CDCl_3_.

Figure S11. The NOESY spectrum of **2** in CDCl_3_

Figure S12. The ^1^H NMR spectrum of **3** in CDCl_3_ (500 MHz).

Figure S13. The ^13^C NMR spectrum of **3** in CDCl_3_ (125 MHz).

Figure S14. The HSQC spectrum of **3** in CDCl_3._

Figure S15. The ^1^H−^1^H COSY spectrum of **3** in CDCl_3._

Figure S16. The HMBC spectrum of **3** in CDCl_3._

Figure S17. The NOESY spectrum of **3** in CDCl_3_

Figure S18. The ^1^H NMR spectrum of **3** in DMSO-*d*_6_ (500 MHz).

Figure S19. The NOESY spectrum of **3** in DMSO-*d*_6._

Figure S20. The ^1^H NMR spectrum of **4** in CDCl_3_ (500 MHz).

Figure S21. The ^13^C NMR spectrum of **4** in CDCl_3_ (125 MHz).

Figure S22. The ^1^H NMR spectrum of **5** in CDCl_3_ (500 MHz).

Figure S23. The ^13^C NMR spectrum of **5** in CDCl_3_ (125 MHz).

Figure S24. The ^1^H NMR spectrum of **6** in CDCl_3_ (500 MHz).

Figure S25. The ^13^C NMR spectrum of **6** in CDCl_3_ (125 MHz).

Figure S26. The ^1^H NMR spectrum of **7** in CDCl_3_ (500 MHz).

Figure S27. The ^13^C NMR spectrum of **7** in CDCl_3_ (125 MHz).

Figure S28. The ^1^H NMR spectrum of **8** in CDCl_3_ (500 MHz).

Figure S29. The ^13^C NMR spectrum of **8** in CDCl_3_ (125 MHz).


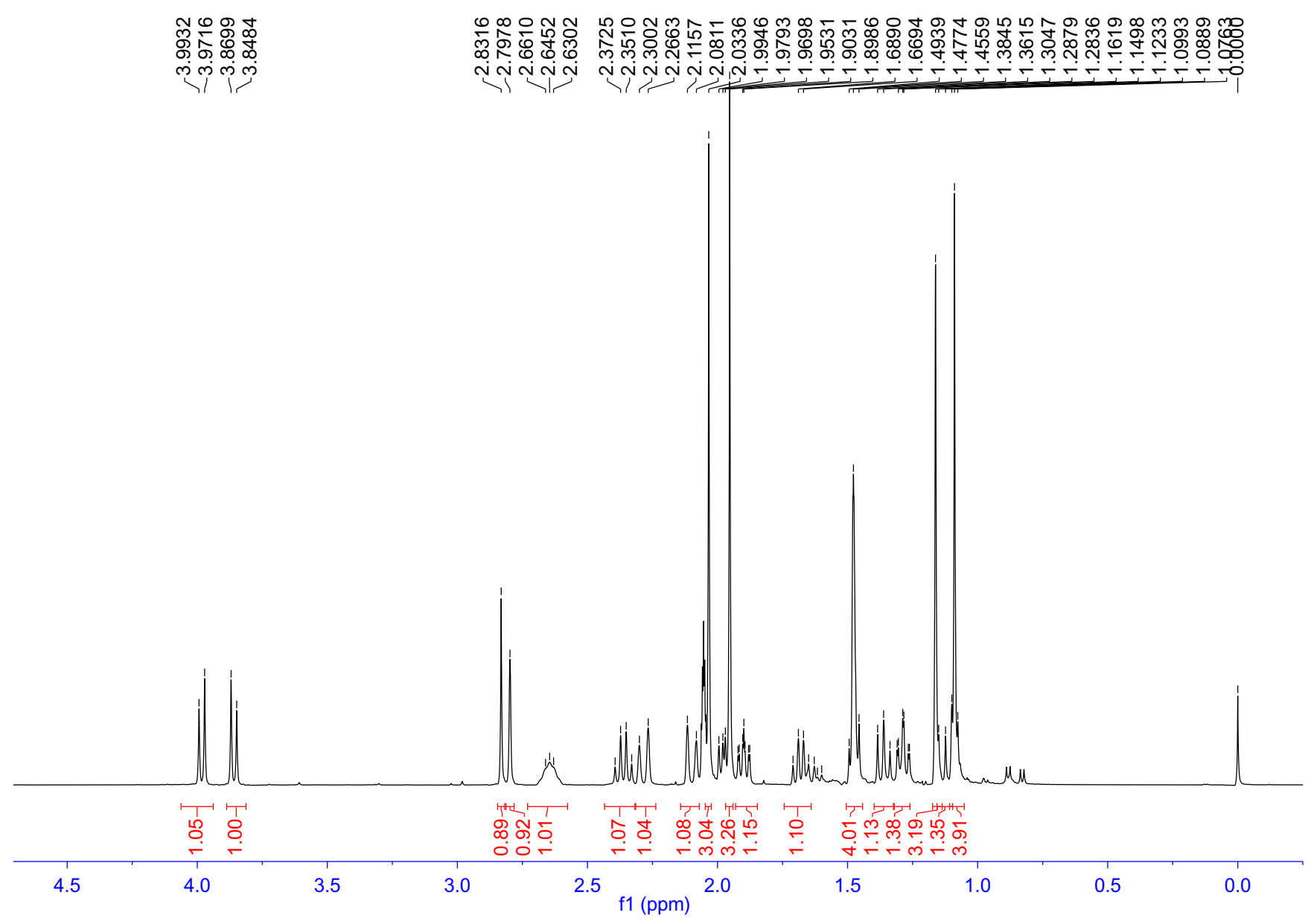


Figure S1. The ^1^H NMR spectrum of **1** in acetone-*d*_6_ (500 MHz).


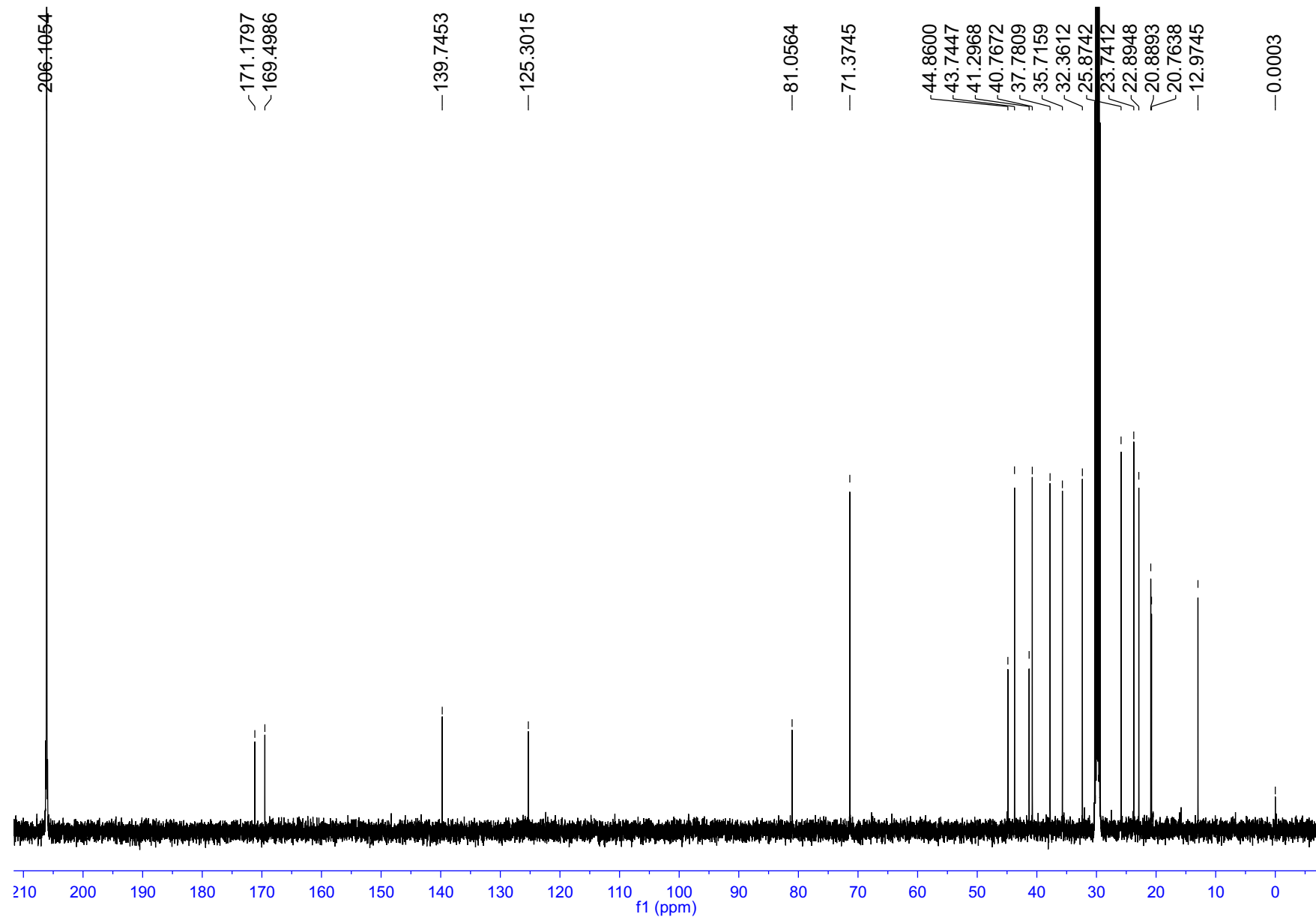


Figure S2. The ^13^C NMR spectrum of **1** in acetone-*d*_6_ (125 MHz).


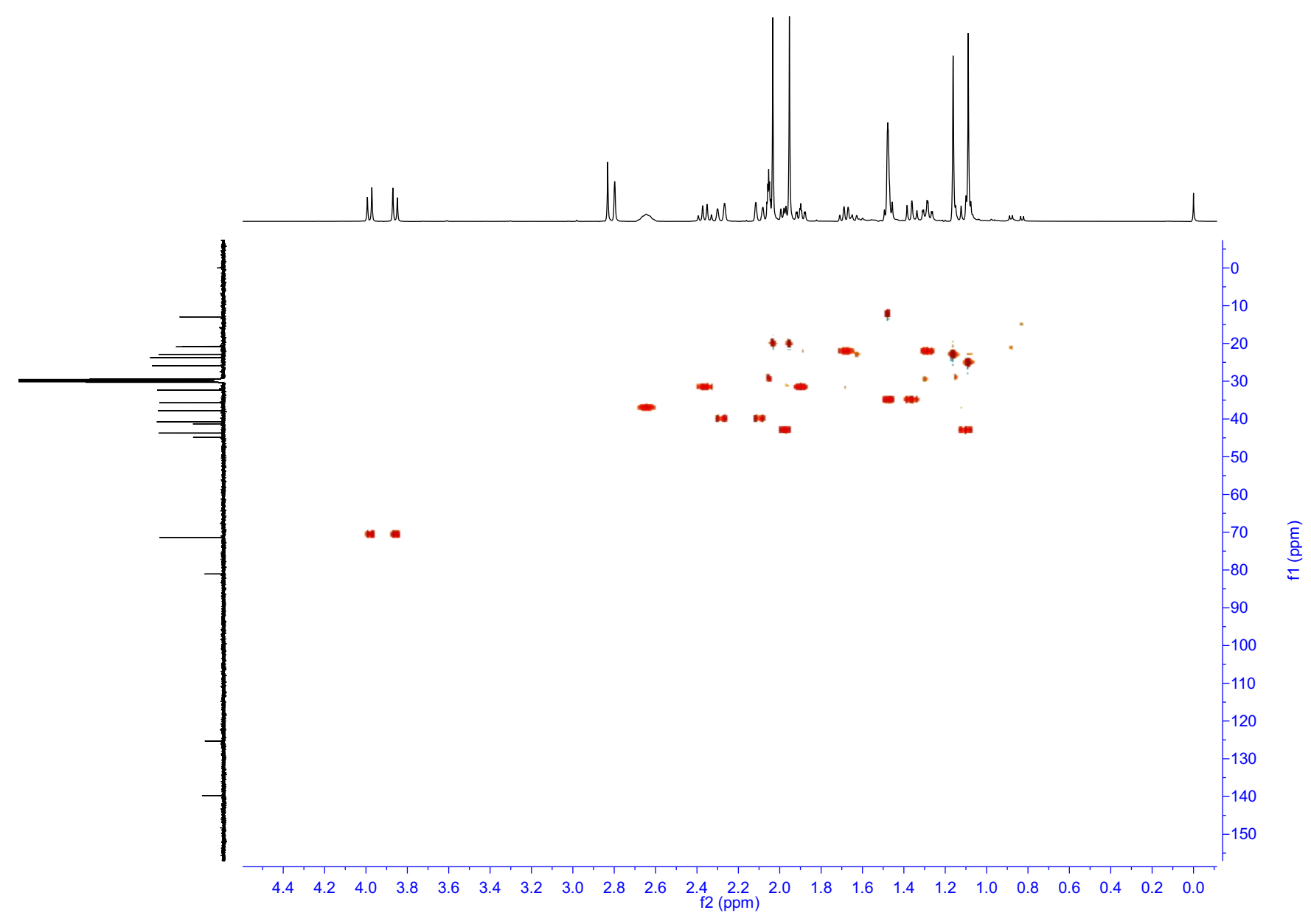


Figure S3. The HSQC spectrum of **1** in acetone-*d*_6_


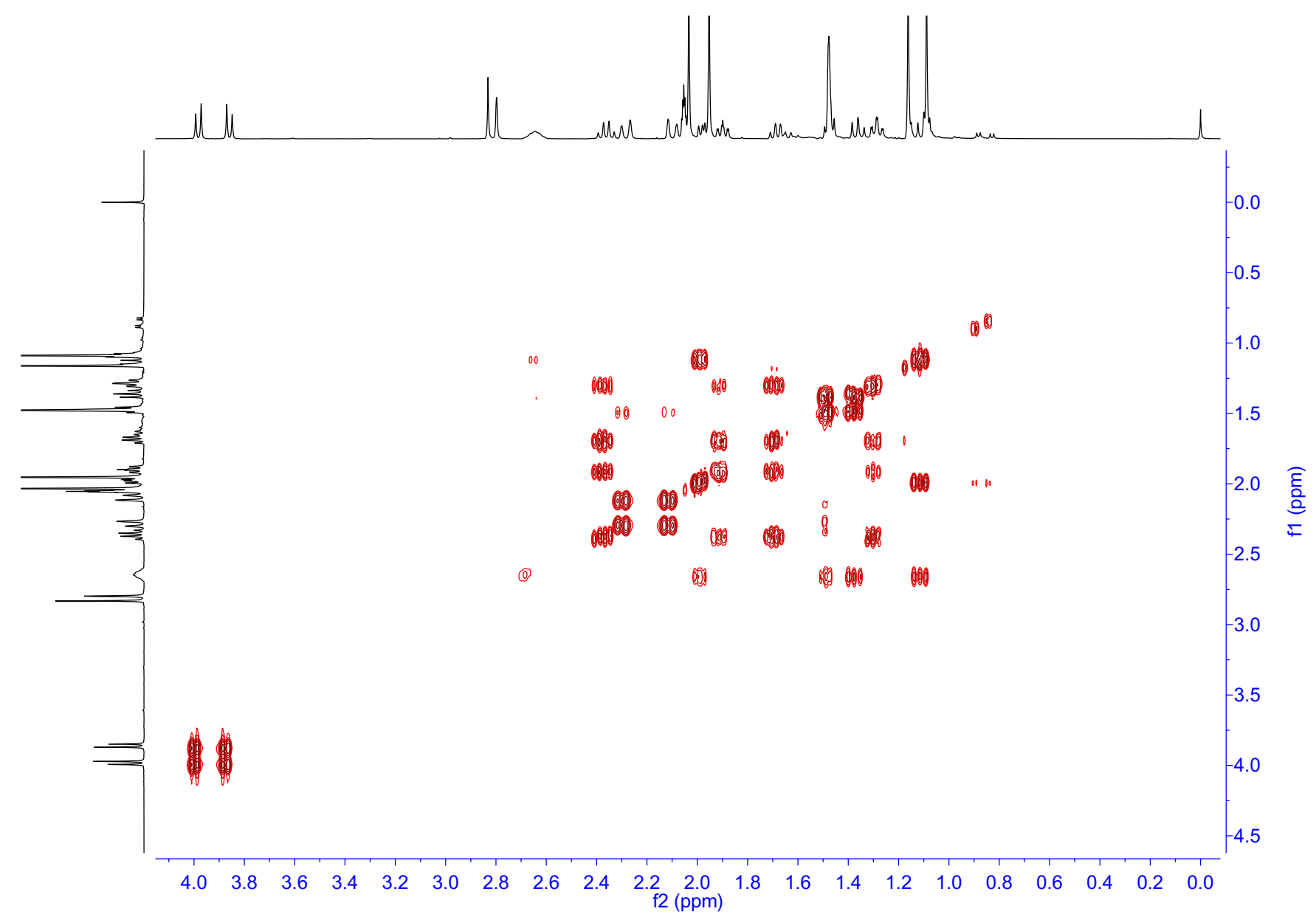


Figure S4. The ^1^H−^1^H COSY spectrum of **1** in acetone-*d*_6._


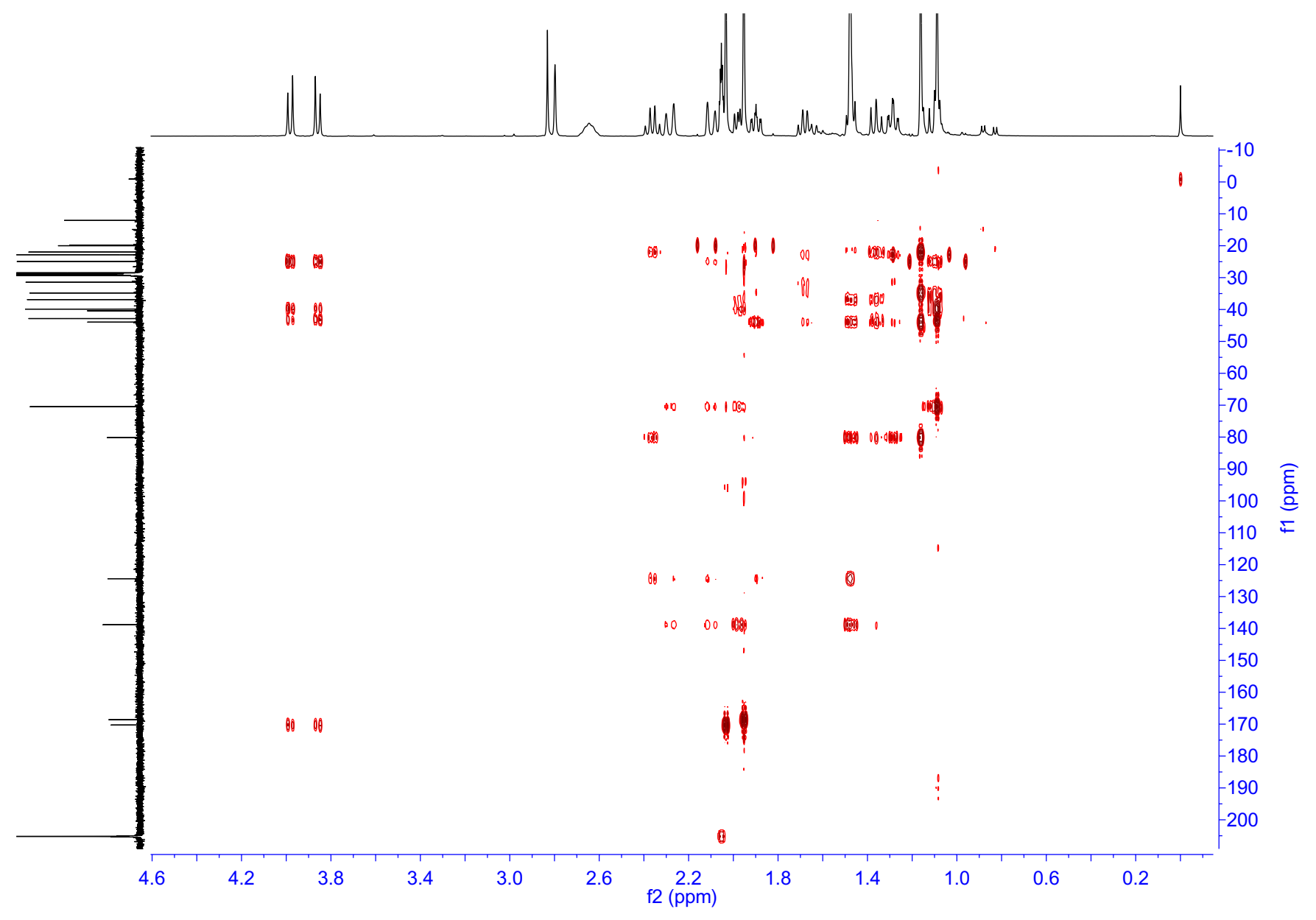


Figure S5. The HMBC spectrum of **1** in acetone-*d*_6._


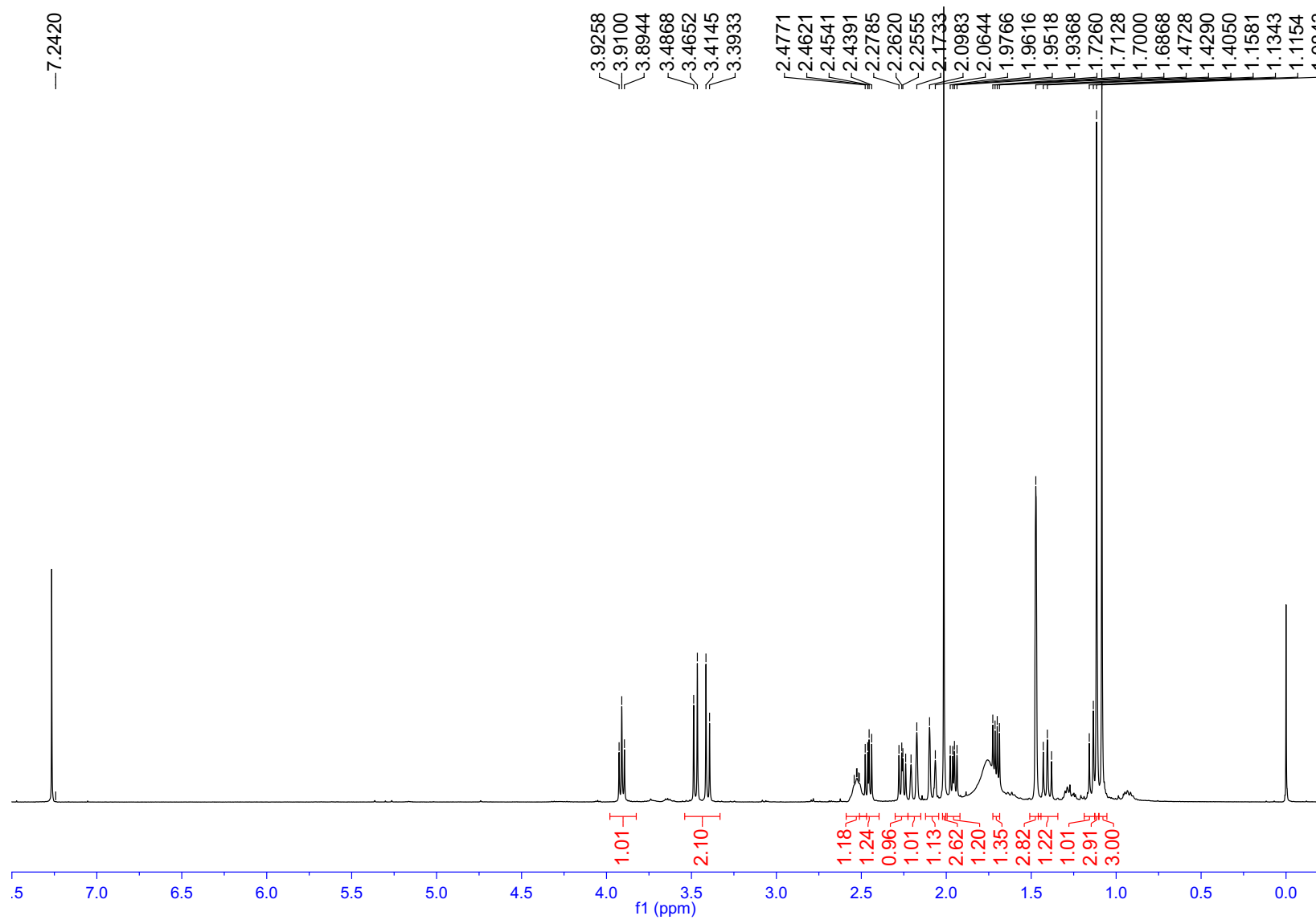


Figure S6. The ^1^H NMR spectrum of **2** in CDCl_3_ (500 MHz).


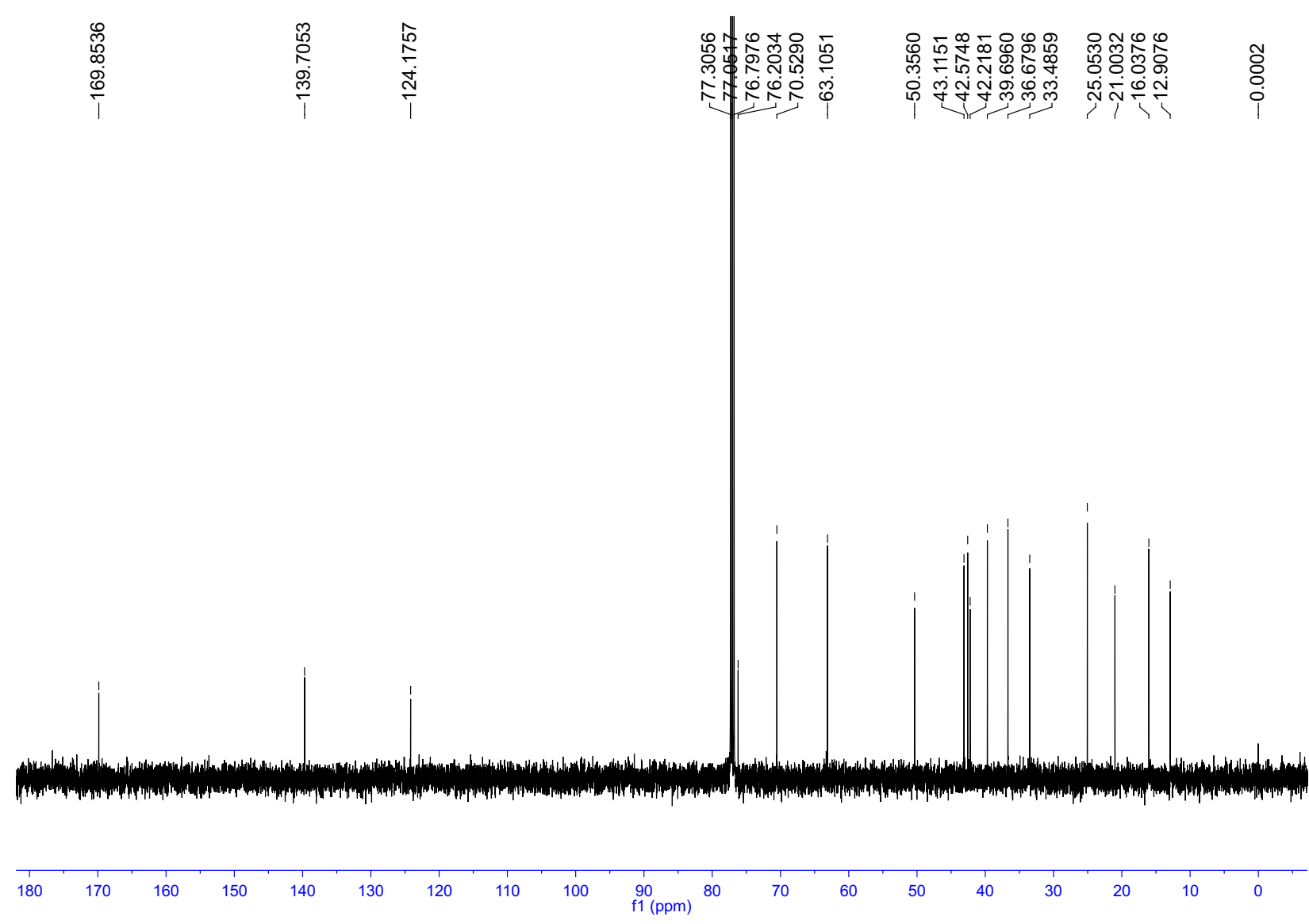


Figure S7. The ^13^C NMR spectrum of **2** in CDCl_3_ (125 MHz).


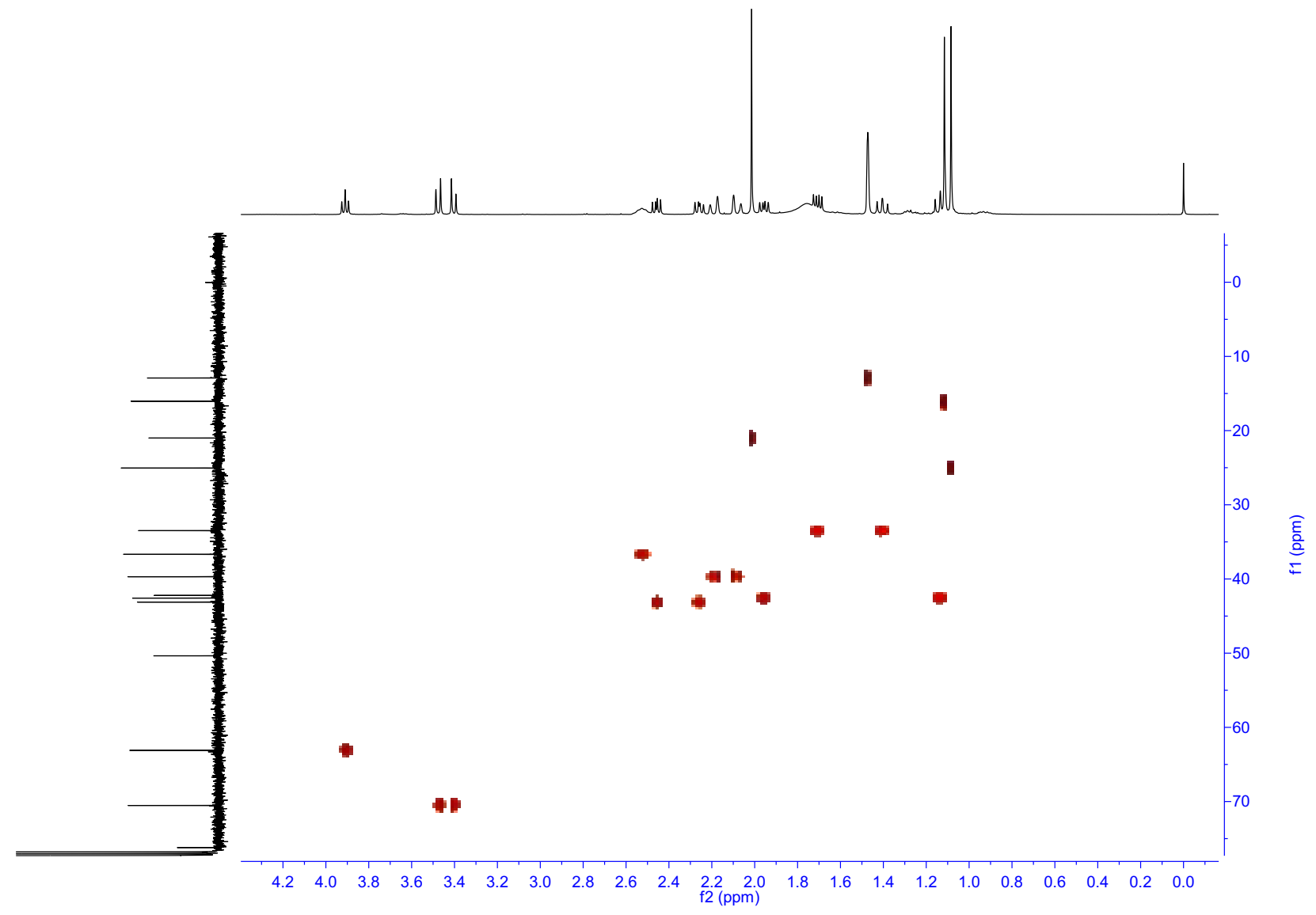


Figure S8. The HSQC spectrum of **2** in a CDCl_3._


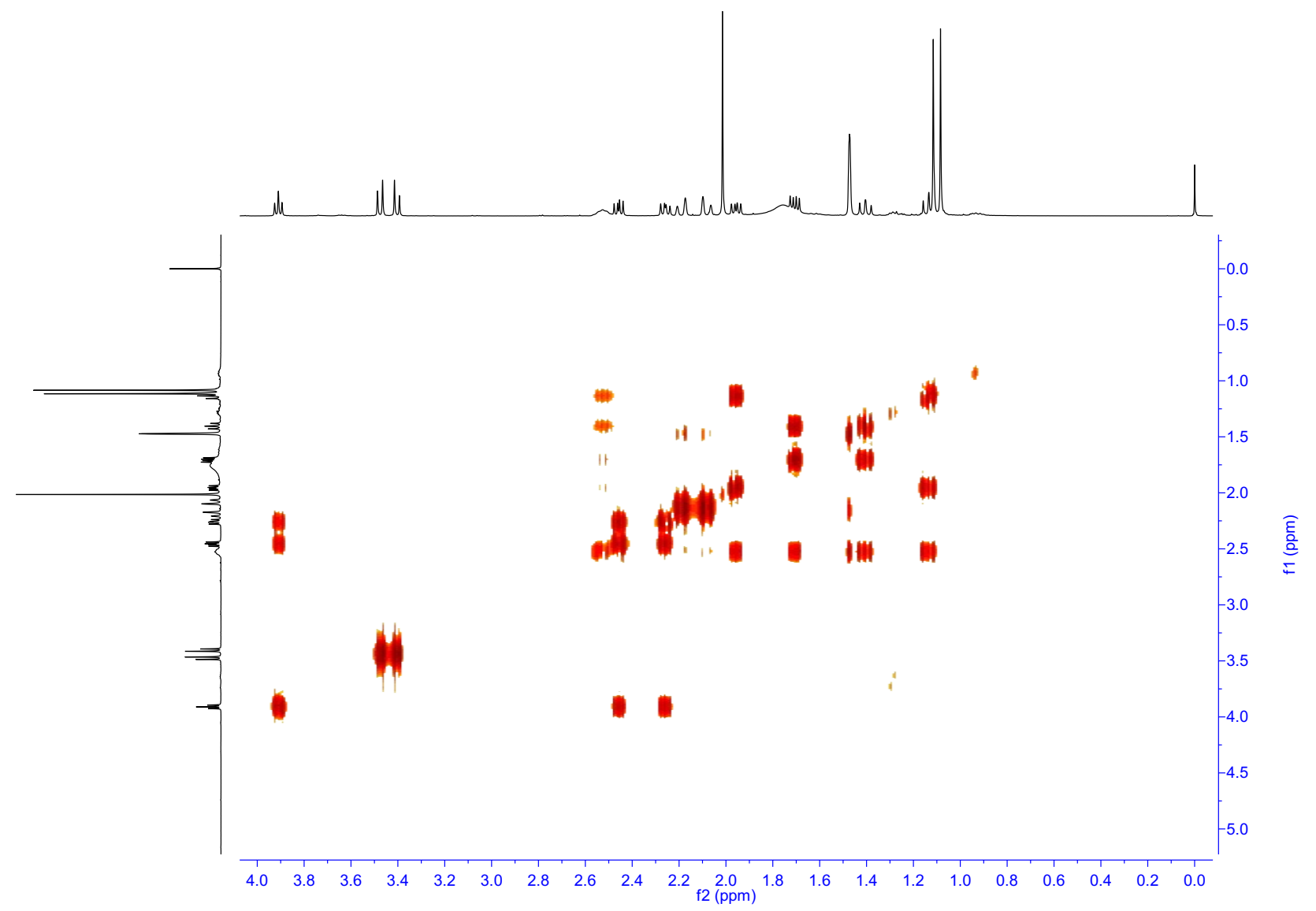
Figure S9. The ^1^H−^1^H COSY spectrum of **2** in CDCl_3._


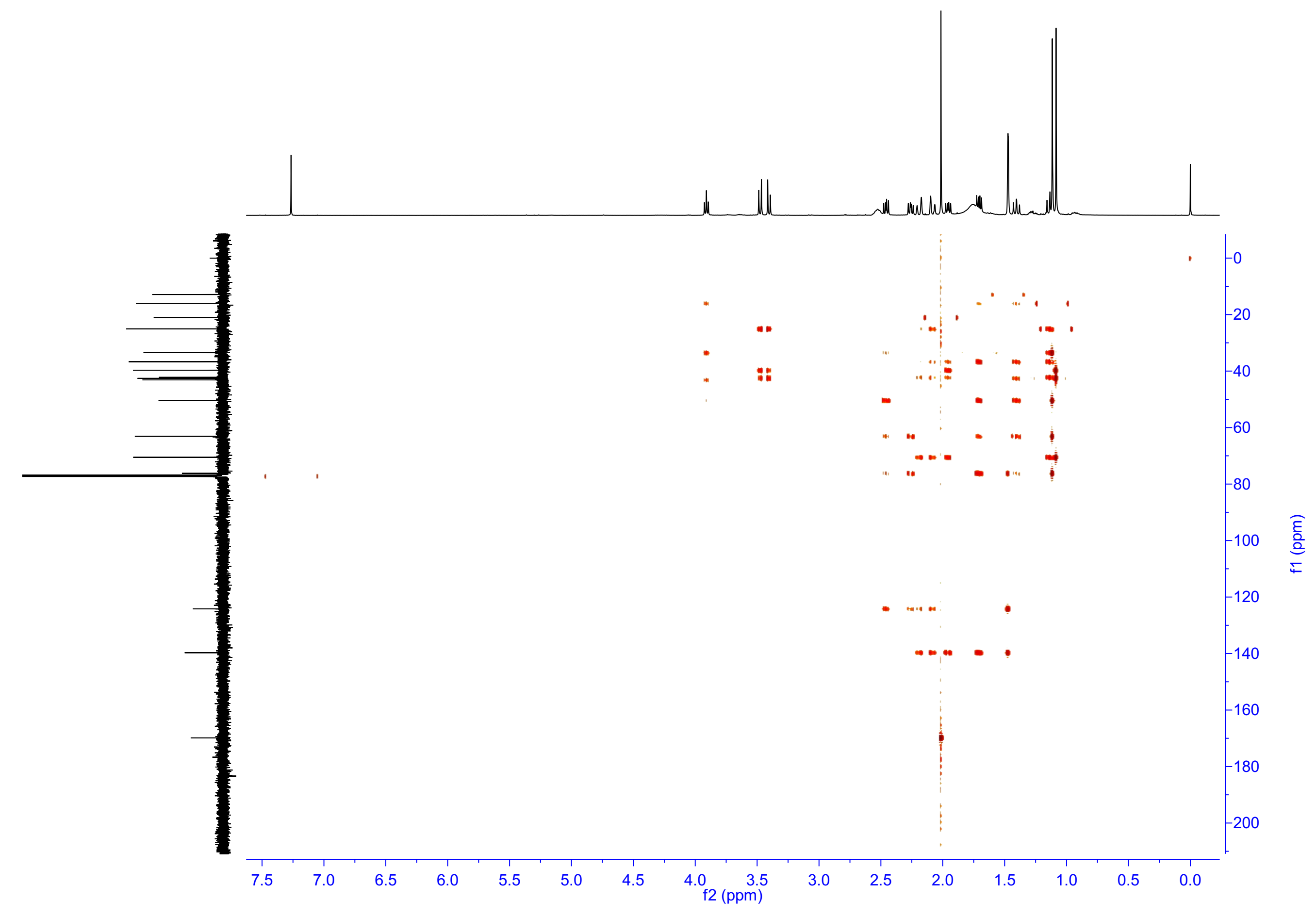


Figure S10.The HMBC spectrum of **2** in CDCl_3_.


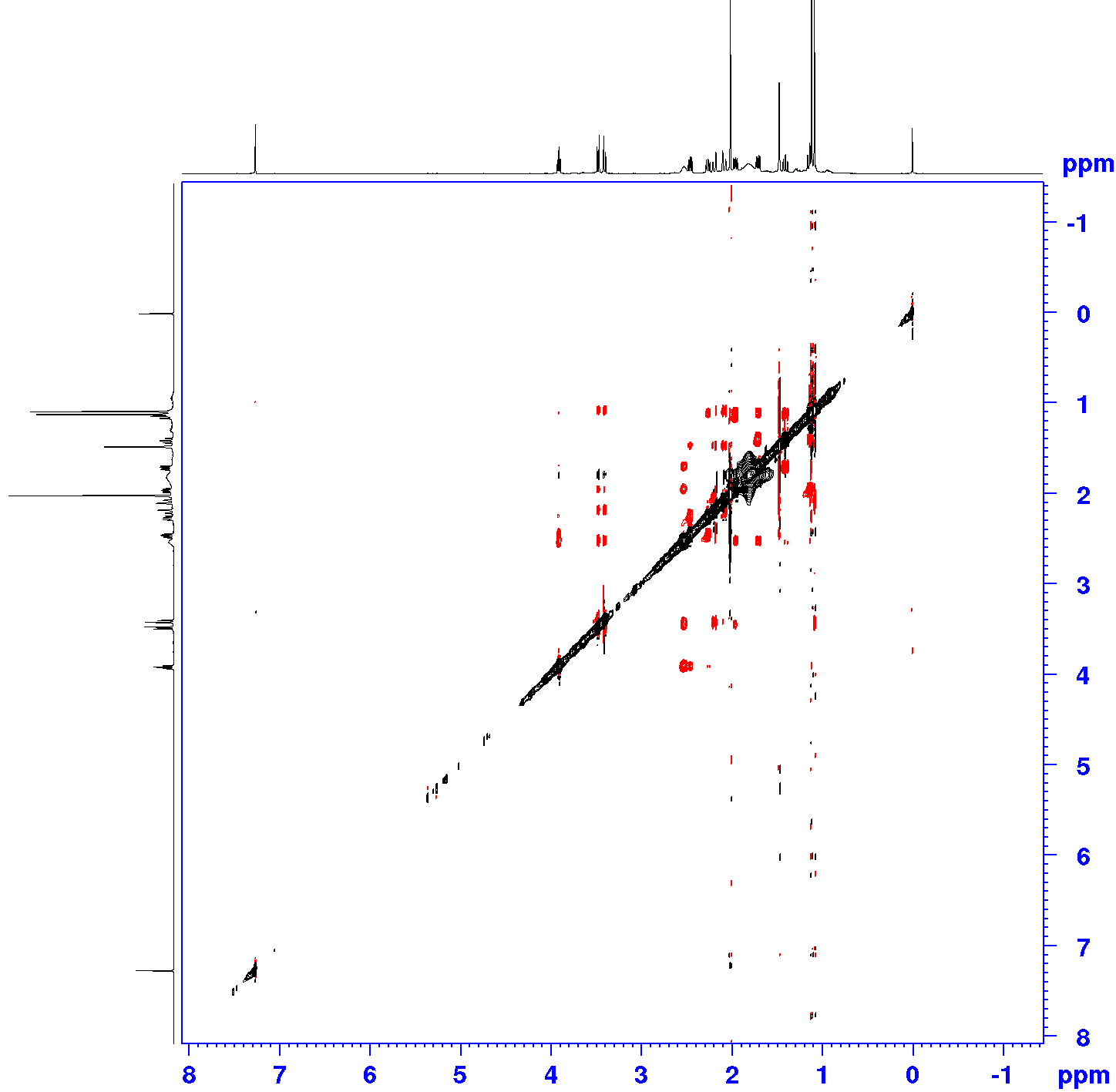


Figure S11. The NOESY spectrum of **2** in CDCl_3_


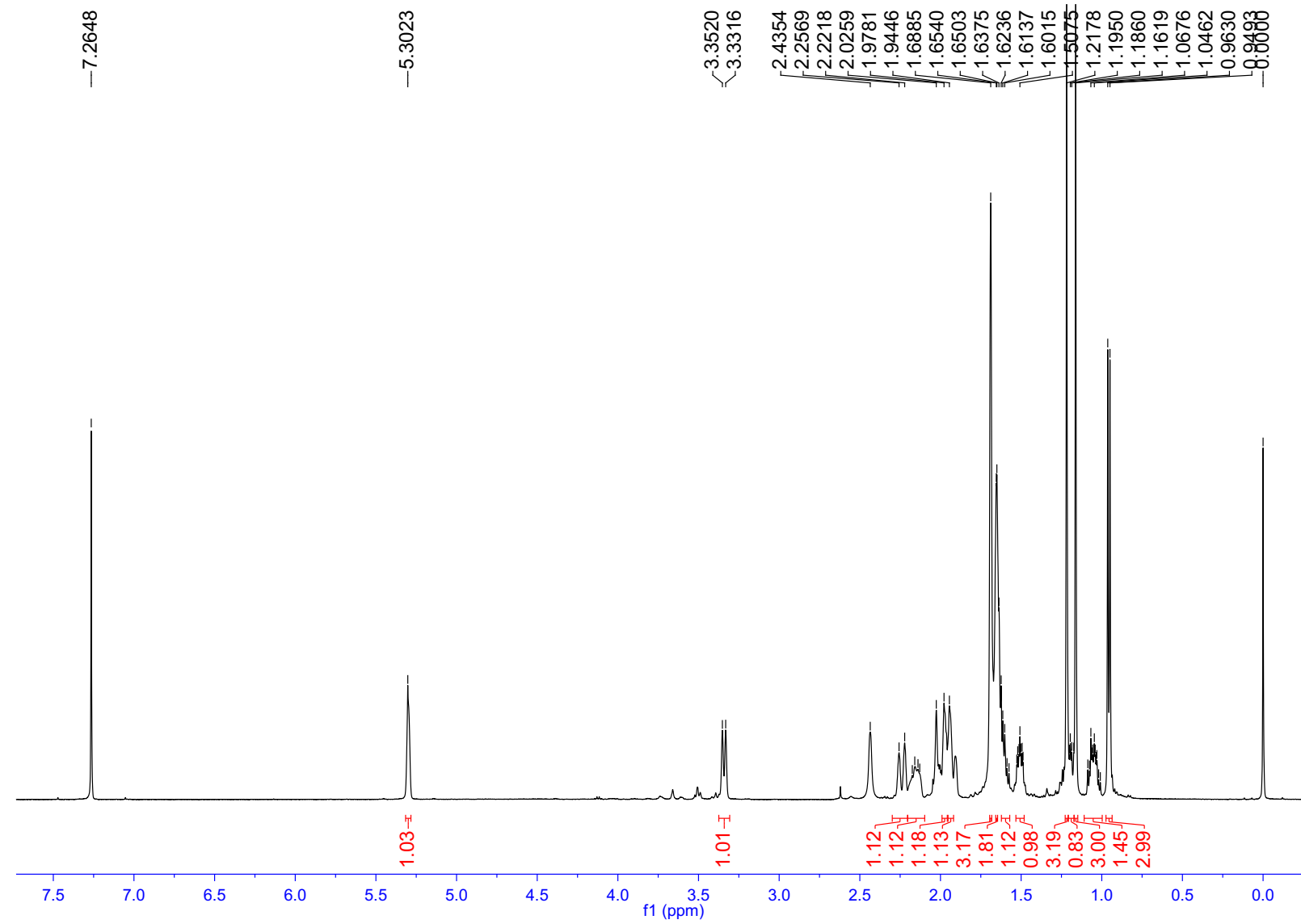


Figure S12. The ^1^H NMR spectrum of **3** in CDCl_3_ (500 MHz).


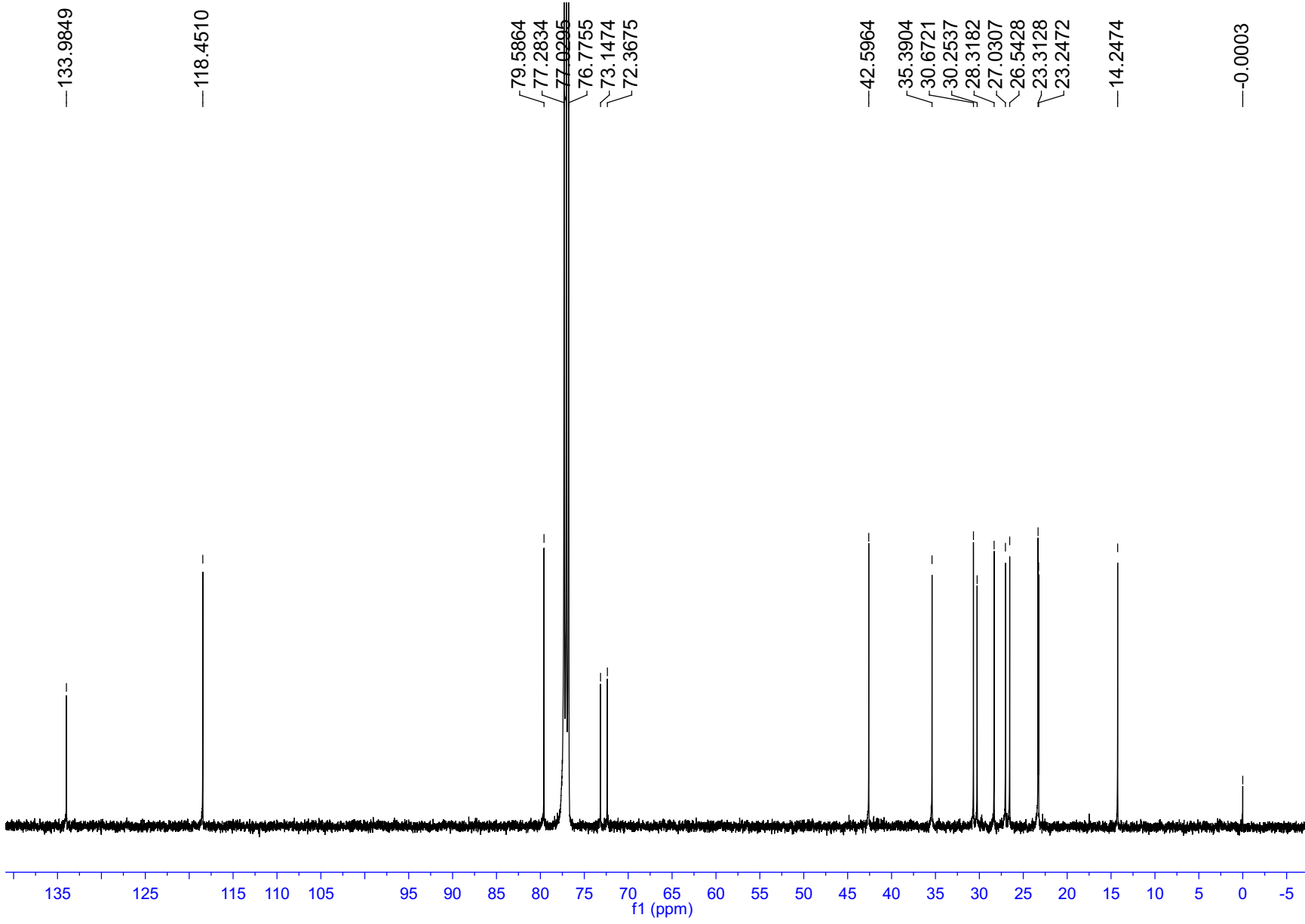


Figure S13. The ^13^C NMR spectrum of **3** in CDCl_3_ (125 MHz).


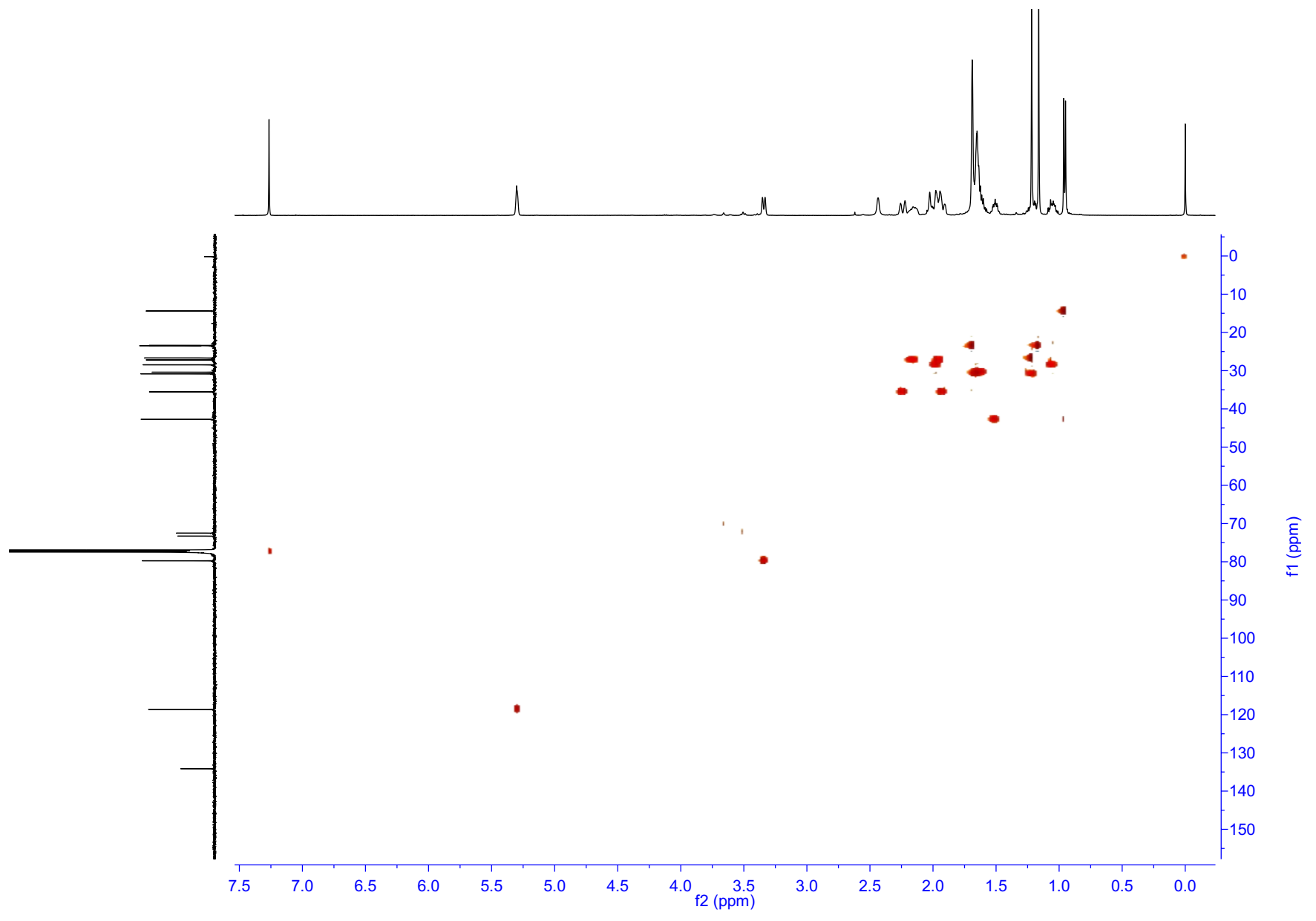


Figure S14. The HSQC spectrum of **3** in CDCl_3._


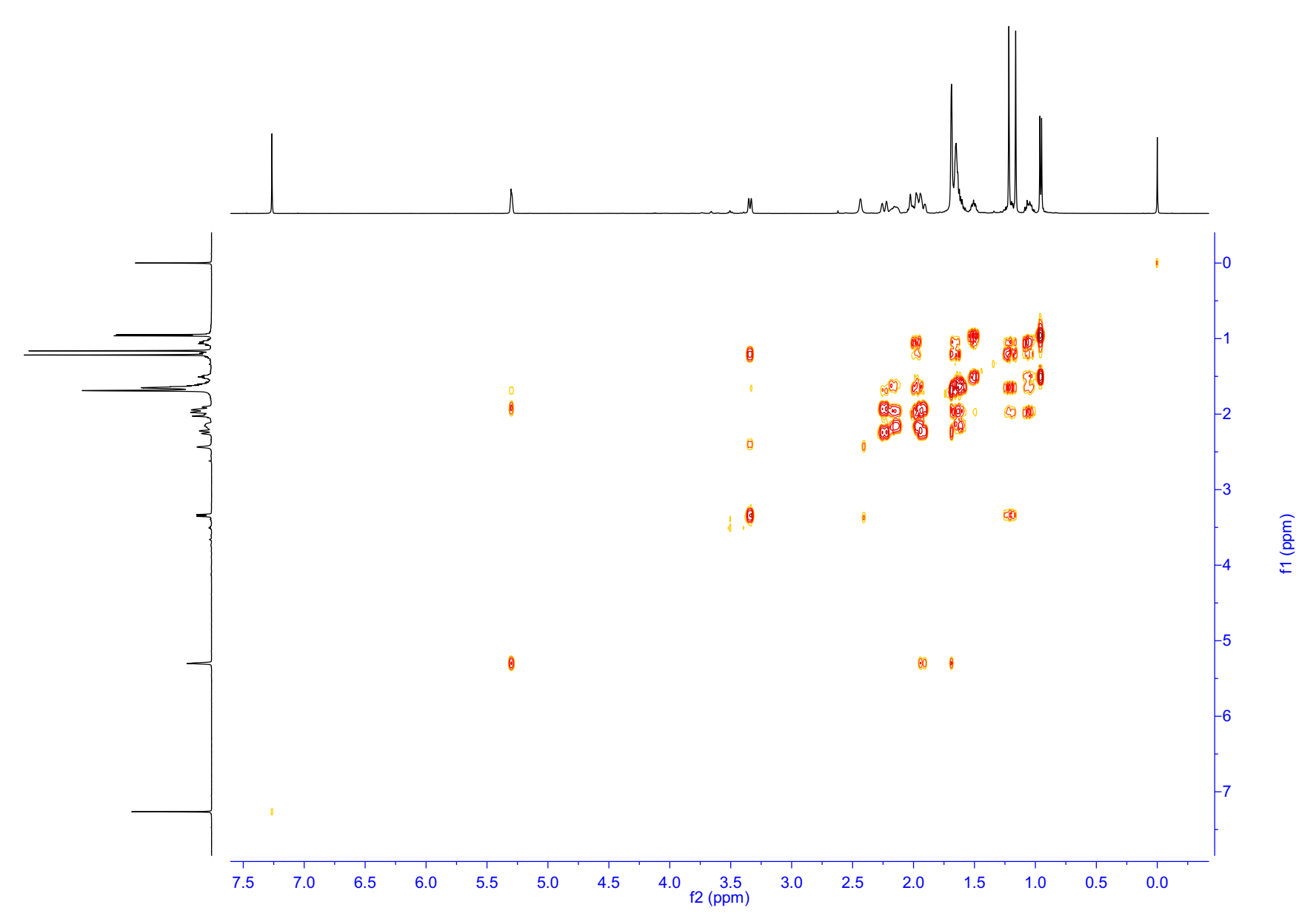


Figure S15. The ^1^H−^1^H COSY spectrum of **3** in CDCl_3._


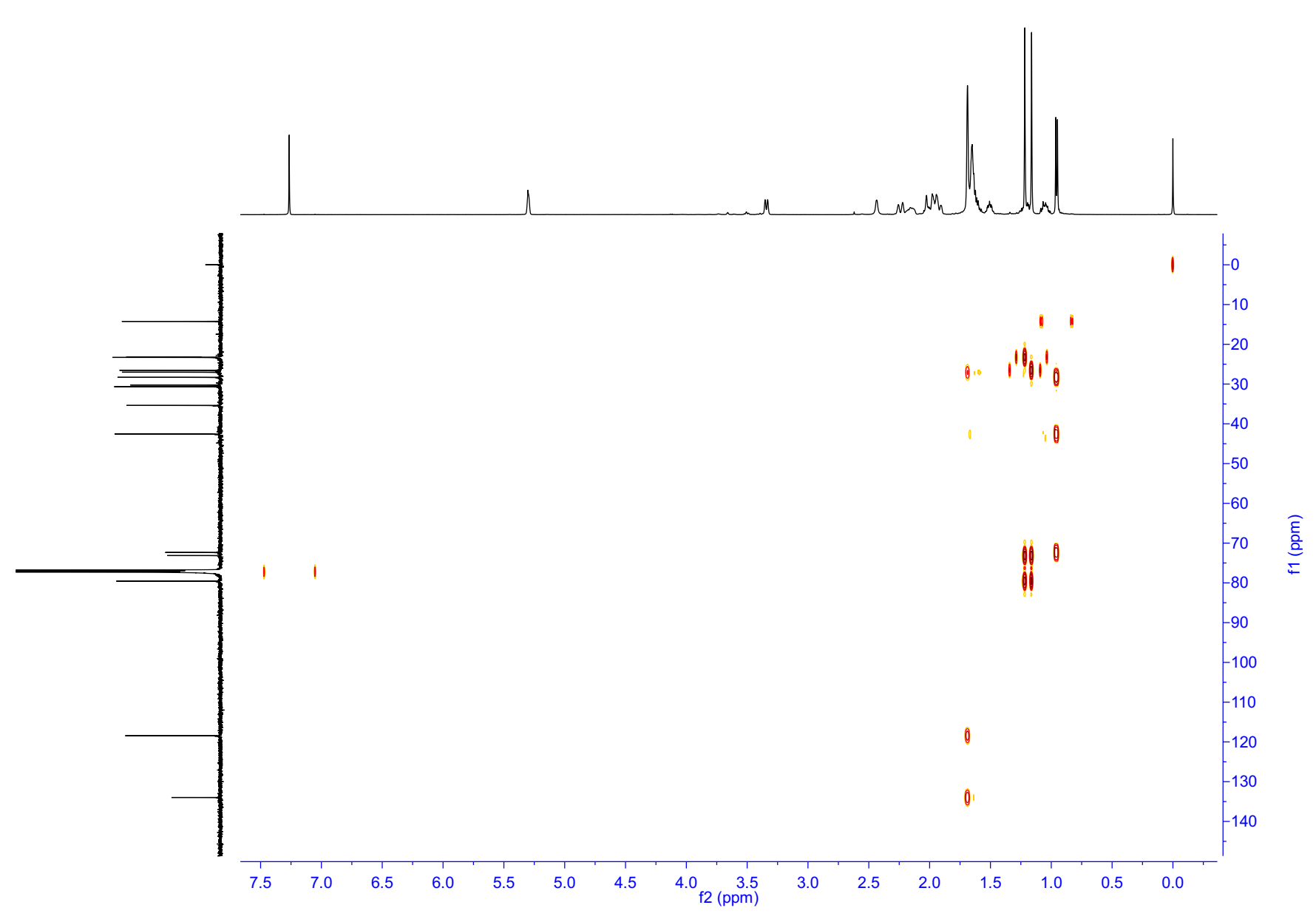


Figure S16. The HMBC spectrum of **3** in CDCl_3._


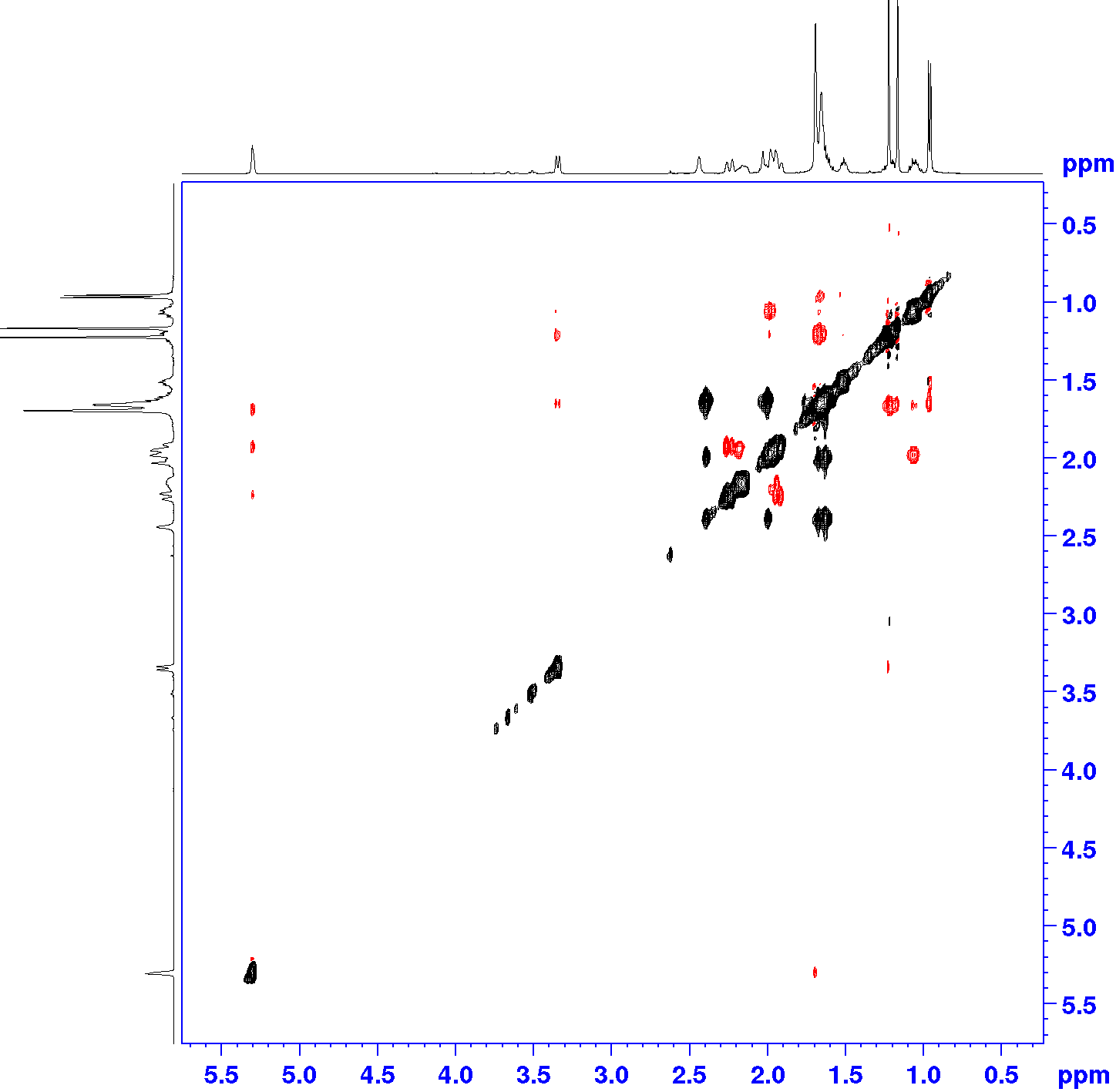


Figure S17. The NOESY spectrum of **3** in CDCl_3_

Figure S18. The ^1^H NMR spectrum of **3** in DMSO-*d*_6_ (500 MHz).


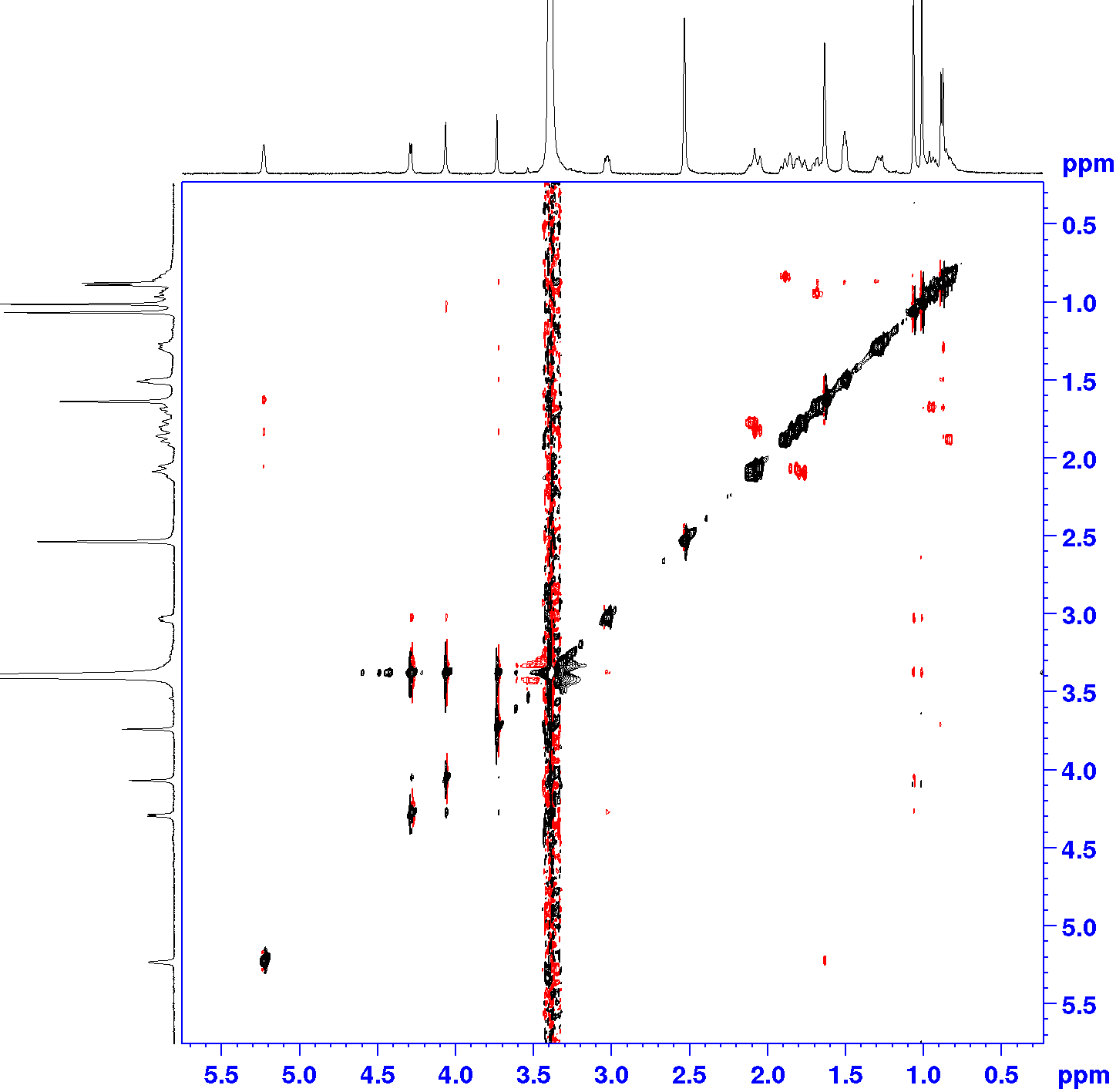


Figure S19. The NOESY spectrum of **3** in DMSO-*d*_6_


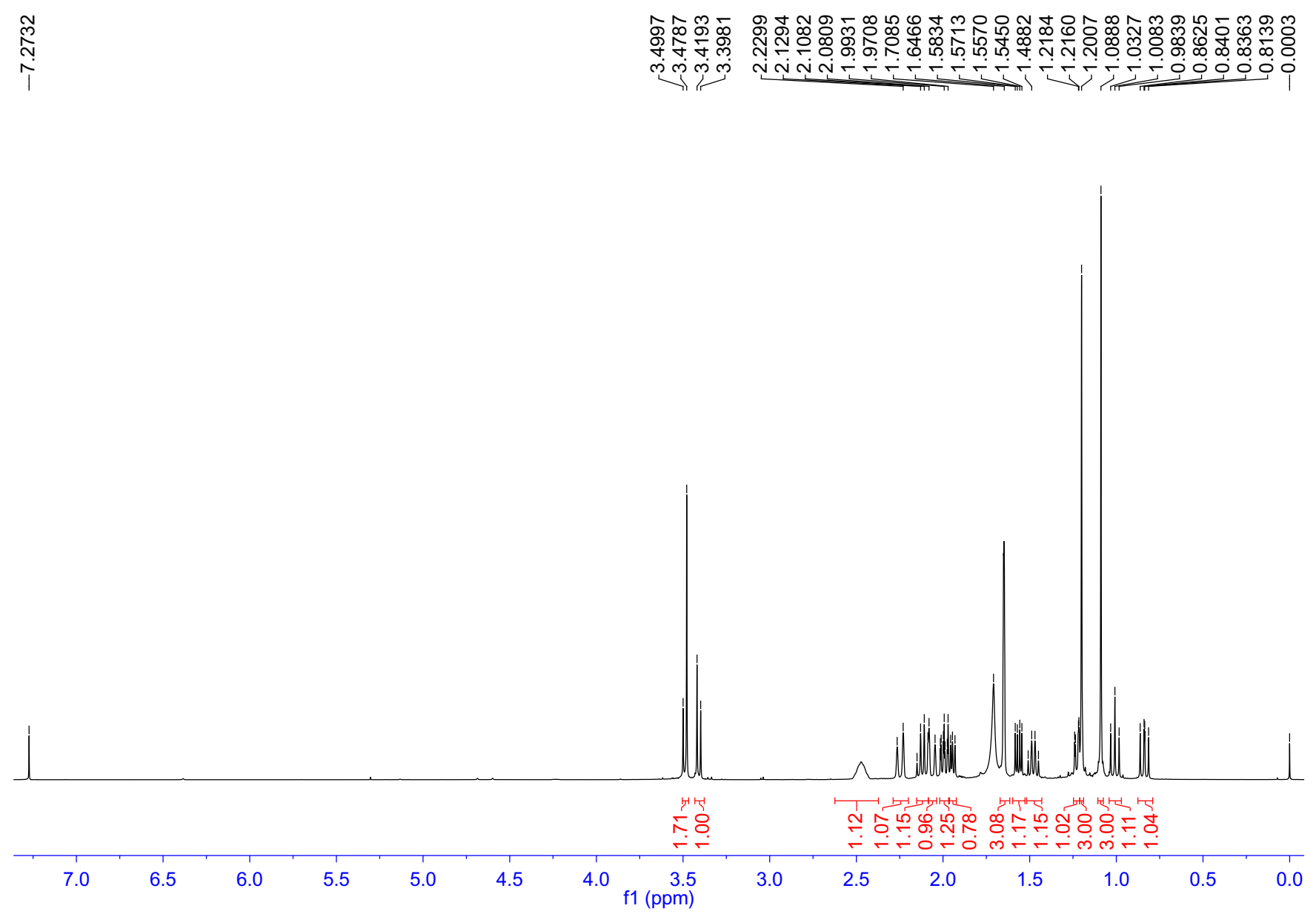


Figure S20. The ^1^H NMR spectrum of **4** in CDCl_3_ (500 MHz).


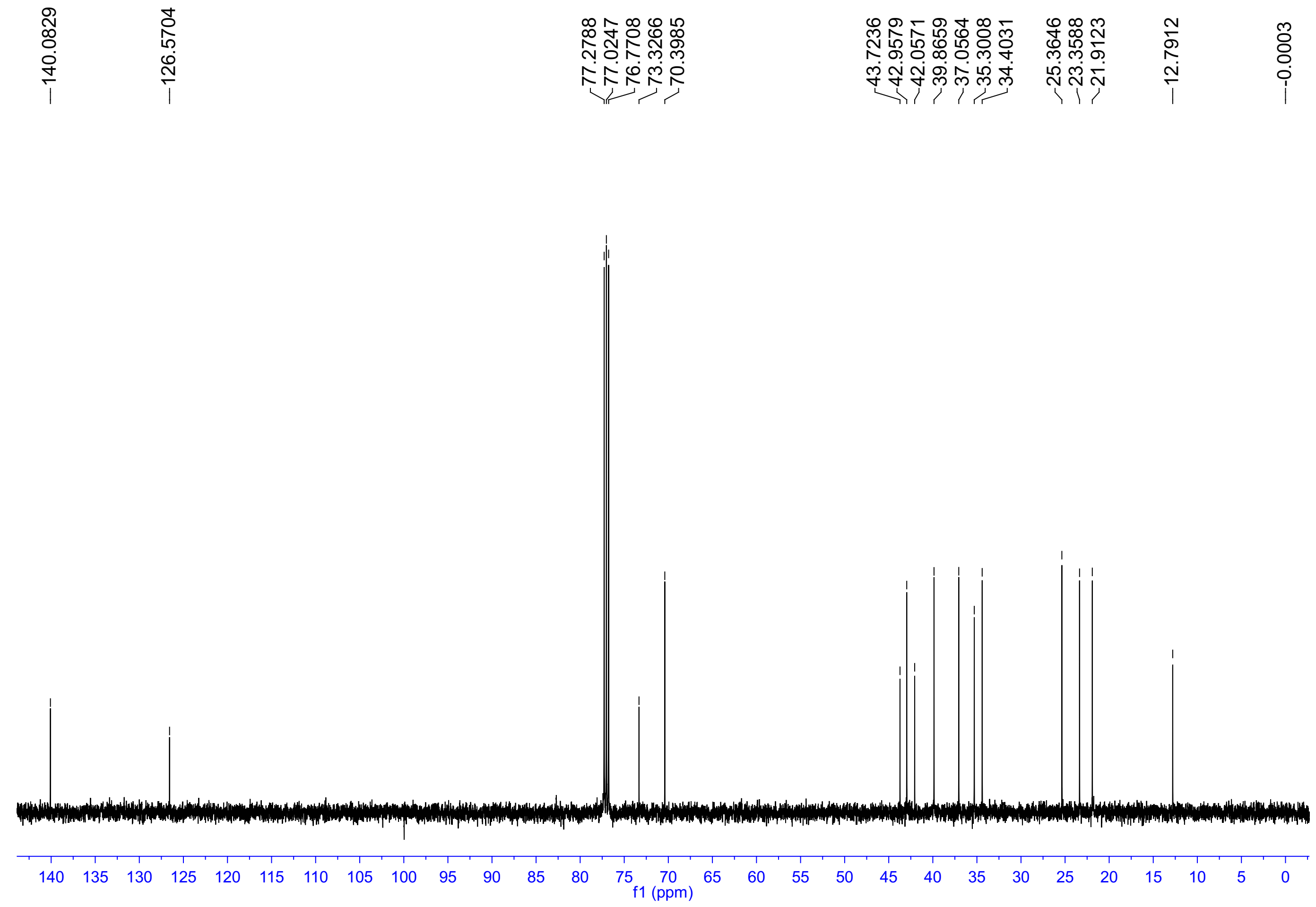


Figure S21. The ^13^C NMR spectrum of **4** in CDCl_3_ (125 MHz).


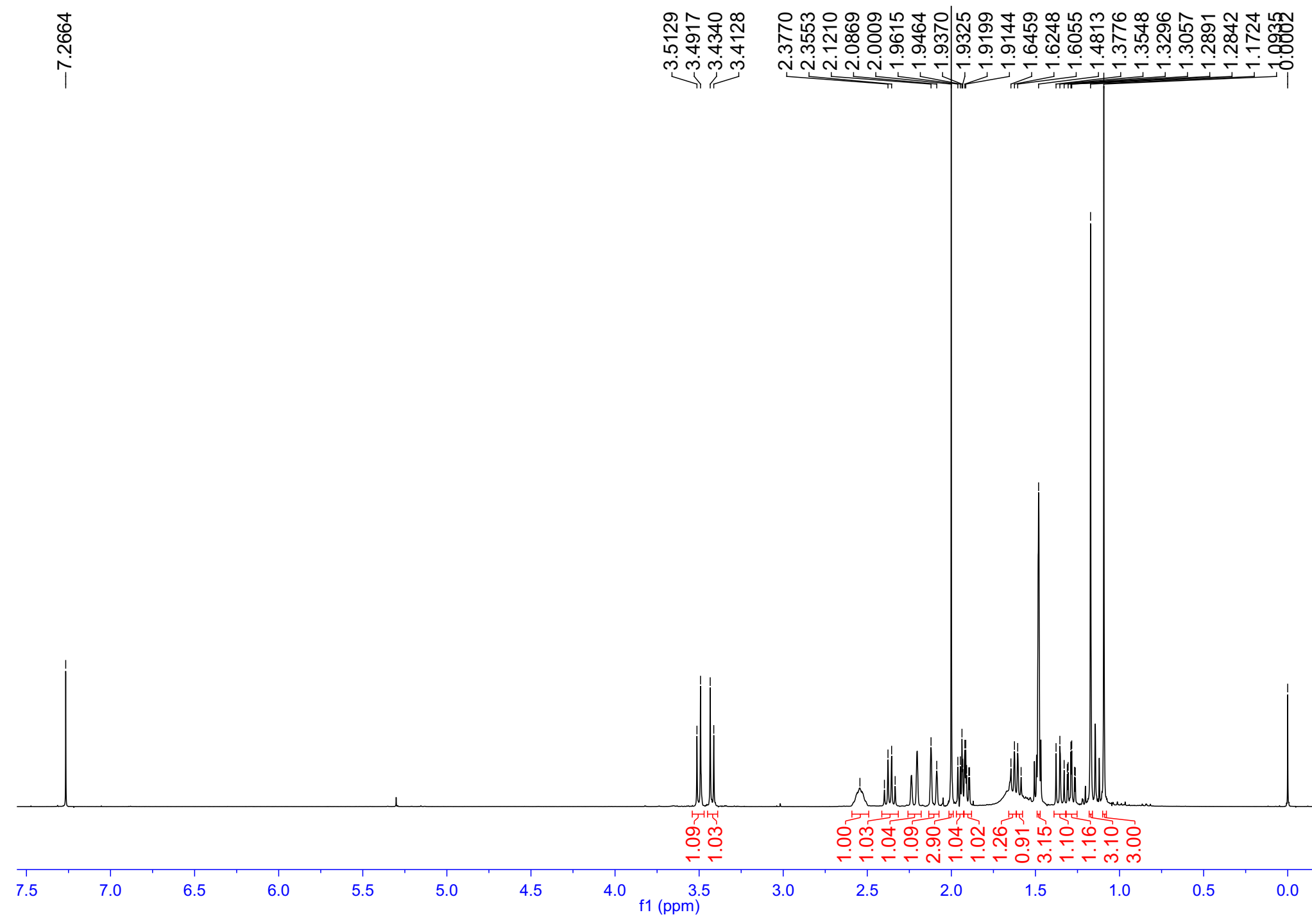


Figure S22. The ^1^H NMR spectrum of **5** in CDCl_3_ (500 MHz).


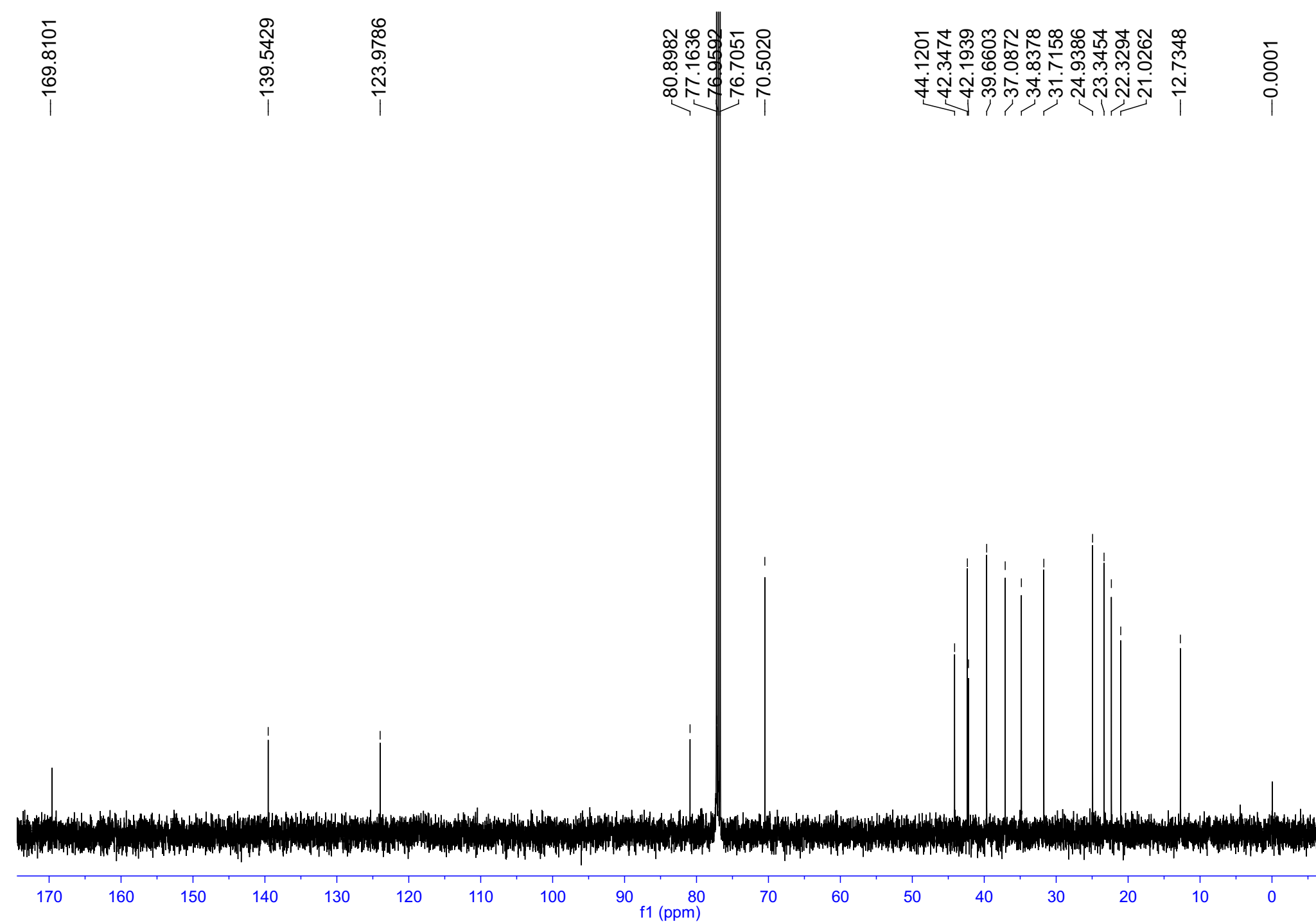


Figure S23. The ^13^C NMR spectrum of **5** in CDCl_3_ (125 MHz).


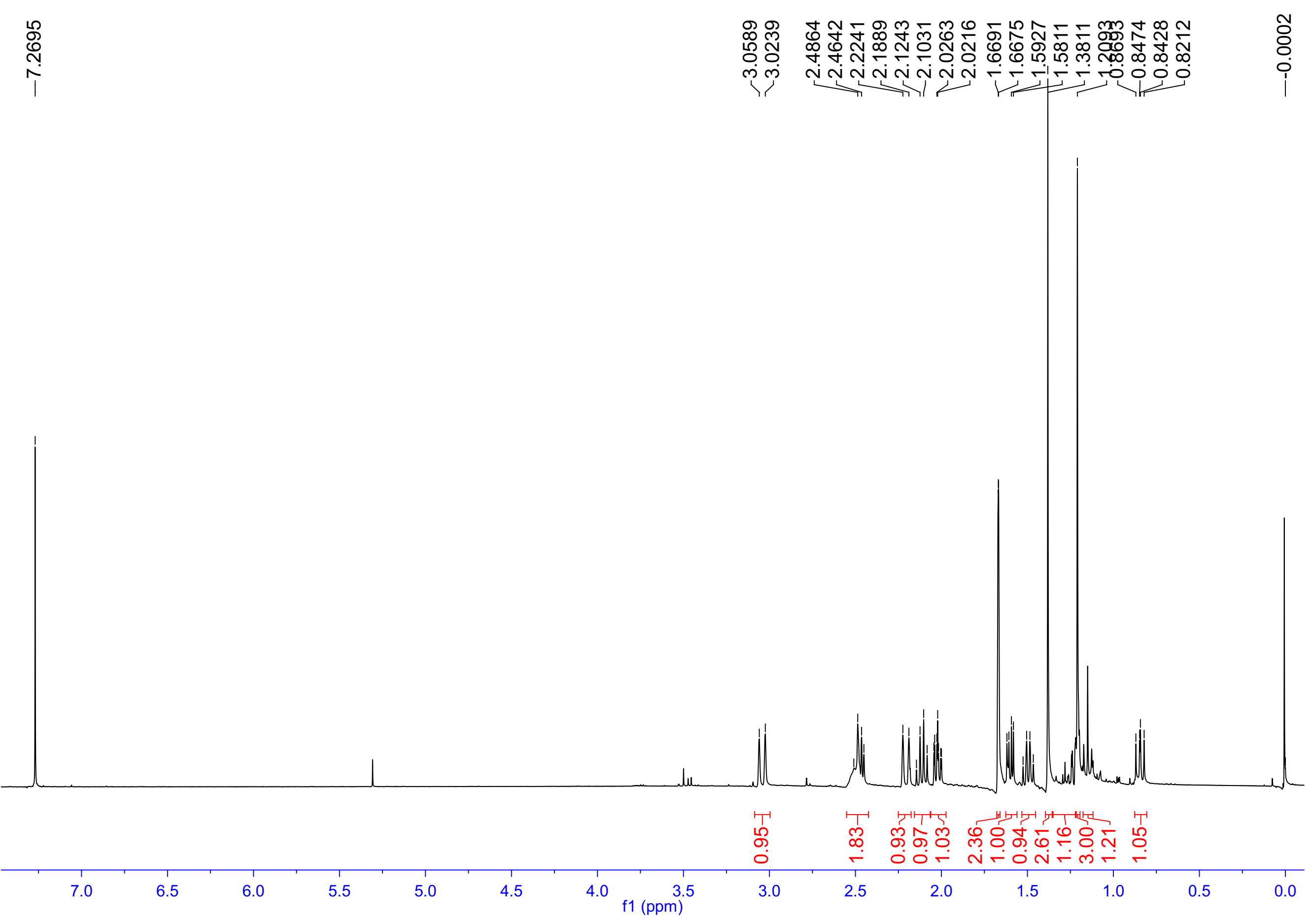


Figure S24. The ^1^H NMR spectrum of **6** in CDCl_3_ (500 MHz).


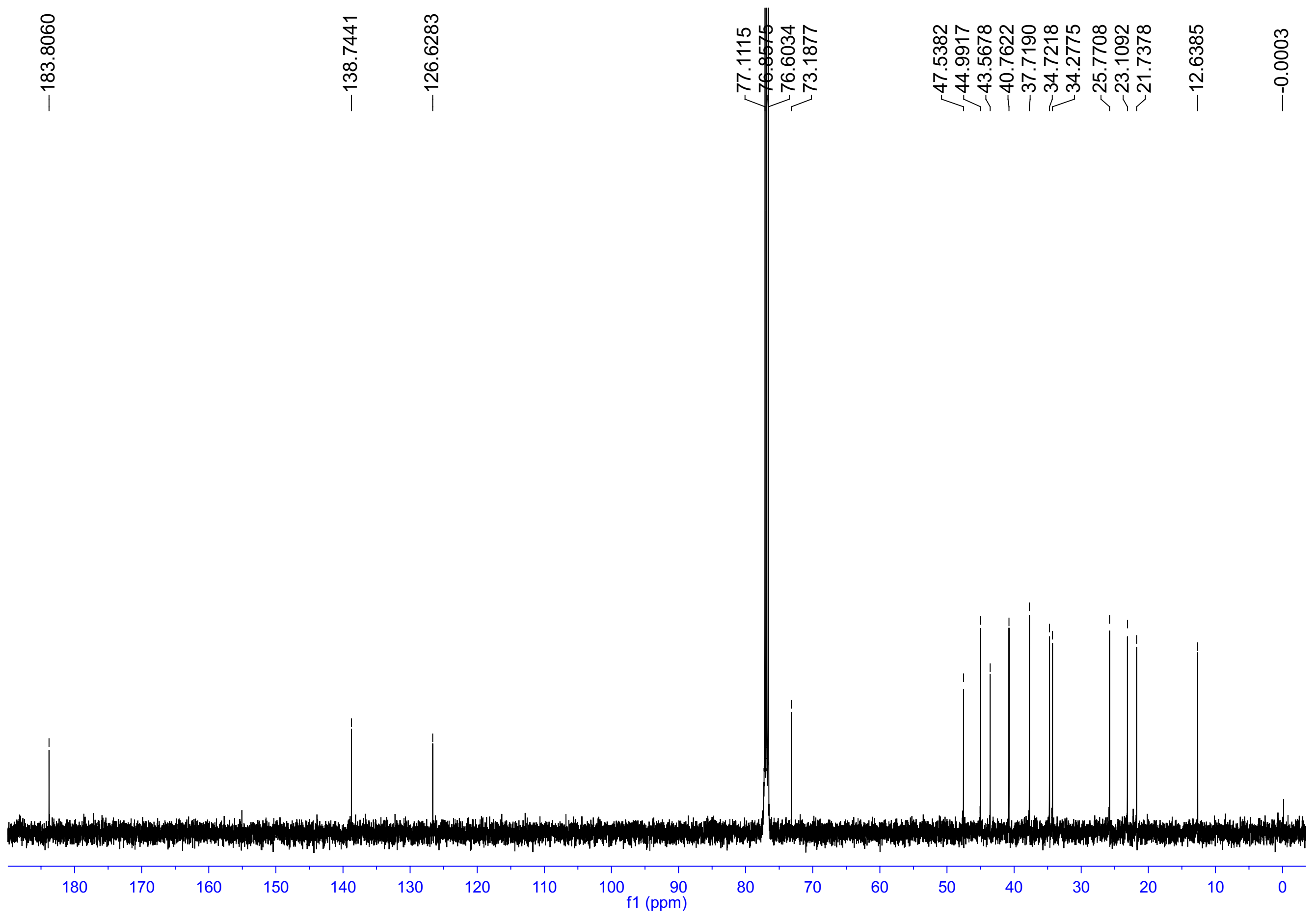
 Figure S25. The ^13^C NMR spectrum of **6** in CDCl_3_ (125 MHz).


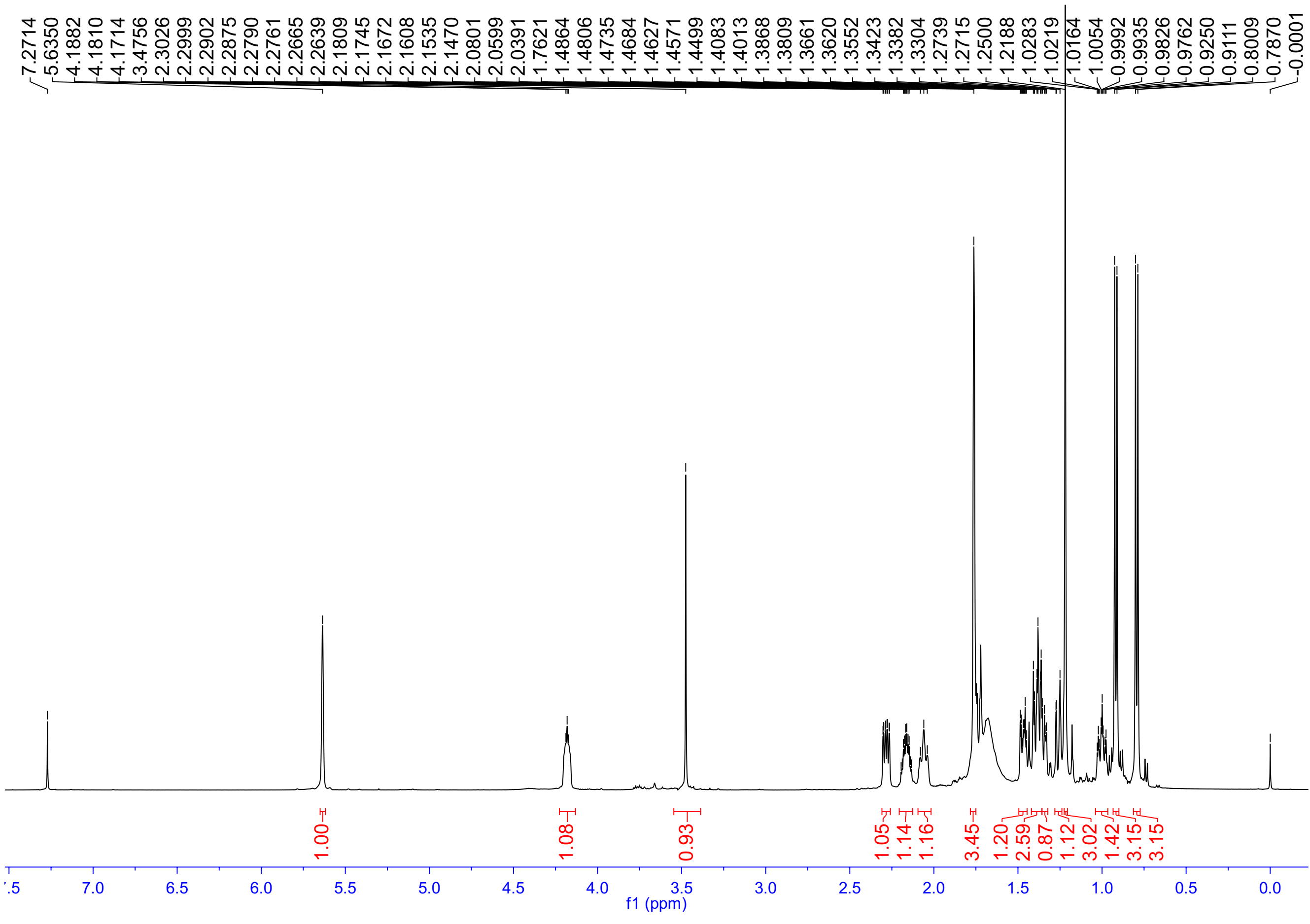


Figure S26. The ^1^H NMR spectrum of **7** in CDCl_3_ (500 MHz).


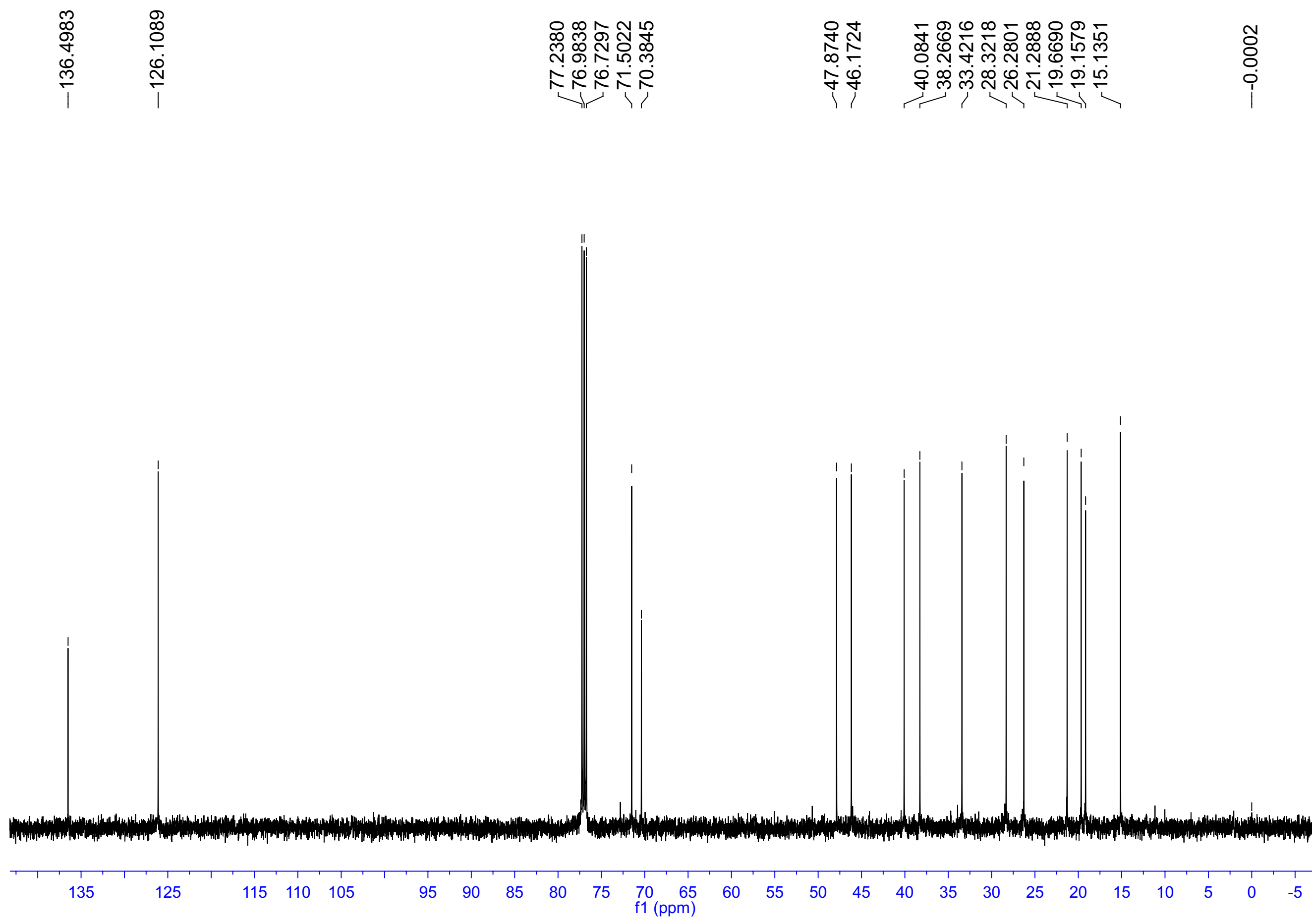


Figure S27. The ^13^C NMR spectrum of **7** in CDCl_3_ (125 MHz).


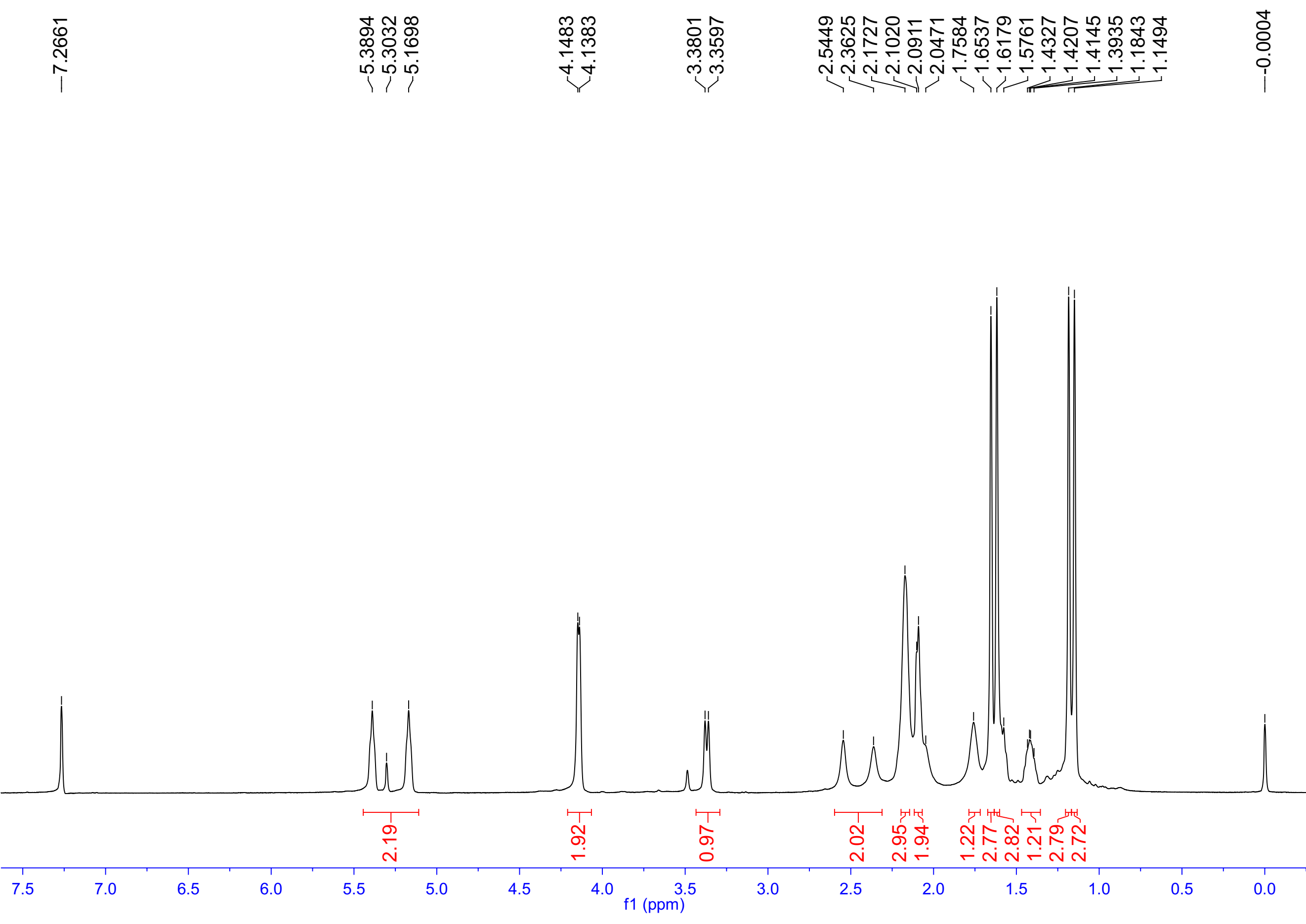


Figure S28. The ^1^H NMR spectrum of **8** in CDCl_3_ (500 MHz).


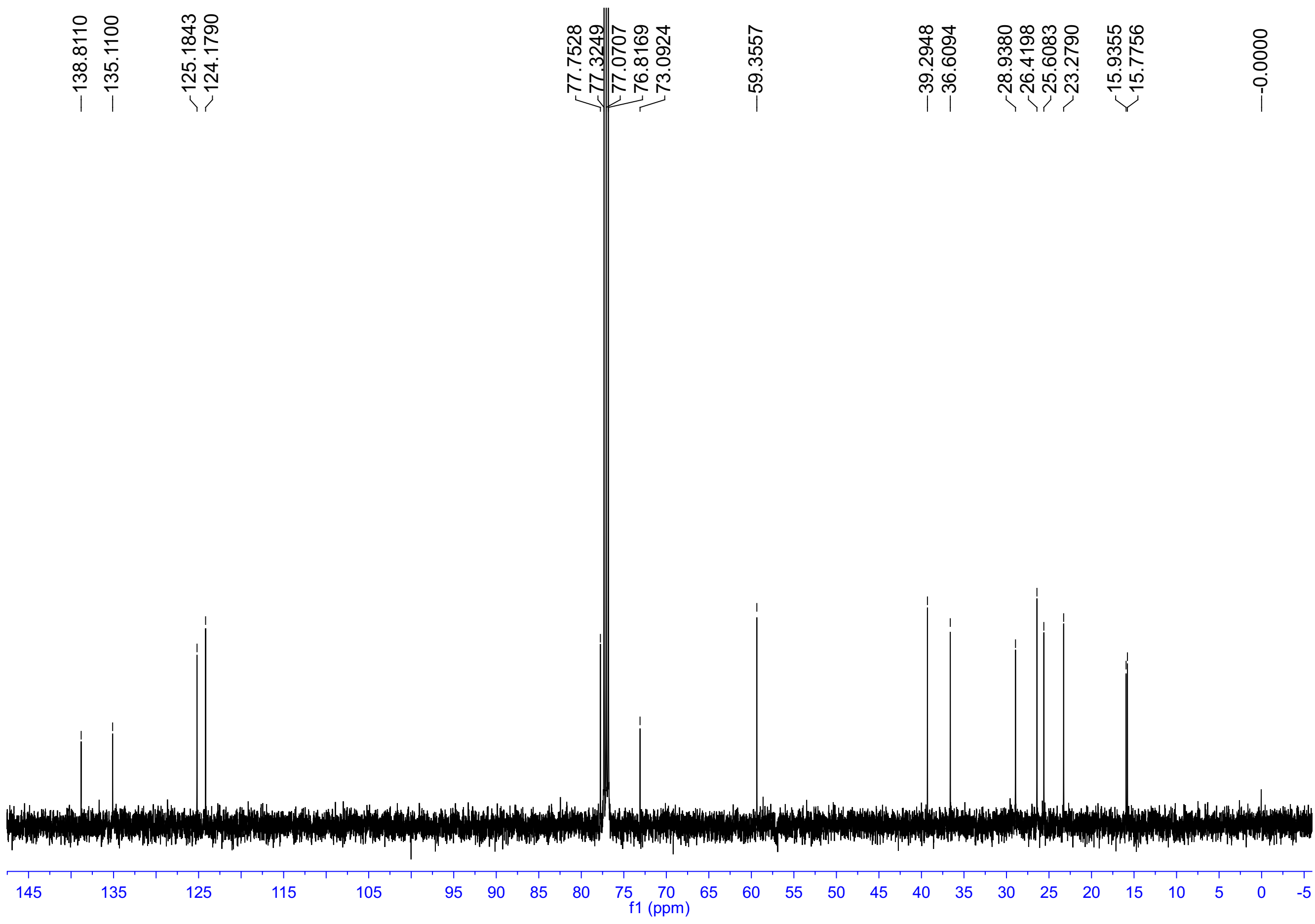


Figure S29. The ^13^C NMR spectrum of **8** in CDCl_3_ (125 MHz).

ITS sequence of *Cryptomarasmius aucubae*

1 GTAACAAGGT TTCCGTAGGT GAACCTGCGG AAGGATCATT ATTGAACTTT GAAACCCTTT

61 GTGGCTTCGT TGGTCTGTAG CTGGCCCTCC TCGGAGGGTA TGTGCTCGCC AGCGTCGCTG

121 CAACTTTCTT TGTCCACCTG TGCACCTTTT GTAGCCTTGG AGCATCTCTC GACGAGGGCC

181 TCTCGGGGTC TTTGCTCGGA TTTGGAGGGC TAGCCTTAGC GCTTCCCTCT GTCGCTCCTA

241 GGCTATGTTA TAACAAACTC TATTGTATGT CTAGAATGTC ATTGCTTTTA TTGGACGCGA

301 GTCCTATAAA ACTTTATACA ACTTTCAACA ACGGATCTCT TGGCTCTCGC ATCGATGAAG

361 AACGCAGCGA AATGCGATAA CTAATGTGAA TTGCAGAATT CAGTGAATCA TCGAGTCTTT

421 GAACGCACCT TGCGCCCTTT GGTATTCCGA AGGGCATGCC TGTTTGAGTG TCATTAACTT

481 CTCAACCTCC CTCACTTTGT TGTGAGCTGG CGGATTGGAT CGTGGGGGCT TGCTGGAGCT

541 CTAGTGGCTT CAGCTCCTCT GAAATGCATT AGCGGAATCC TCCTTGAACT GTGAGGCTTG

601 CTGCAGCTGT GATAATATCT ACGGTTGCTG GCTTGTAGTG AGATTGCTAT GGGTTTTGAA

661 GGGATGCCTC TGGCGCCCTT TGCGTTCTCT TCGAAAGGAG AGATACCTGT CCTTAGCGCT

721 TGCGTACCTT TGAGTTCGAG TTGGCTAGTT CAGCTTCTAA CCGTCTTGTA ACGAGACAAT

781 TATTGACCAT TTGACCTCAA ATCAGGTAGG ACTACCCGCT GAACTTAAGC ATATCA
